# Subjective time perception in dementia: a behavioural and neuroanatomical analysis

**DOI:** 10.1093/braincomms/fcaf496

**Published:** 2025-12-30

**Authors:** Maï-Carmen Requena-Komuro, Jessica Jiang, Elia Benhamou, Harri Sivasathiaseelan, Jeremy C S Johnson, Anthipa Chokesuwattanaskul, Annabel Nelson, Chris J D Hardy, Jason D Warren

**Affiliations:** Dementia Research Centre, UCL Queen Square Institute of Neurology, University College London, London WC1N 3AR, UK; University Medical Center Hamburg-Eppendorf, Institute of Systems Neuroscience, Hamburg 20246, Germany; Dementia Research Centre, UCL Queen Square Institute of Neurology, University College London, London WC1N 3AR, UK; Dementia Research Centre, UCL Queen Square Institute of Neurology, University College London, London WC1N 3AR, UK; Dementia Research Centre, UCL Queen Square Institute of Neurology, University College London, London WC1N 3AR, UK; Dementia Research Centre, UCL Queen Square Institute of Neurology, University College London, London WC1N 3AR, UK; Basic & Clinical Neuroscience, School of Neuroscience, King’s College London, London SE5 9RT, UK; Dementia Research Centre, UCL Queen Square Institute of Neurology, University College London, London WC1N 3AR, UK; Cognitive Clinical and Computational Neuroscience Excellence Center, Faculty of Medicine, Chulalongkorn University, Bangkok 10330, Thailand; Dementia Research Centre, UCL Queen Square Institute of Neurology, University College London, London WC1N 3AR, UK; Dementia Research Centre, UCL Queen Square Institute of Neurology, University College London, London WC1N 3AR, UK; Dementia Research Centre, UCL Queen Square Institute of Neurology, University College London, London WC1N 3AR, UK

**Keywords:** temporal bisection task, emotion, Alzheimer’s disease, frontotemporal dementia, voxel-based morphometry

## Abstract

Subjective time perception—the modulation of elapsed clock time by sensory, homeostatic and psychological factors—is fundamental to how we experience the world. People with Alzheimer’s disease and frontotemporal dementia often exhibit clinically relevant symptoms of altered temporal awareness but these are poorly understood. Here we addressed this issue in a cross-sectional, case-control study of 60 patients representing all major Alzheimer (*n* = 24) and frontotemporal (*n* = 36) syndromes [mean age 68.8 (range 48–77 years); 28% female] and 24 cognitively well age-matched controls [age 69.4 (6.5) years; 50% female]. Subjective duration perception was assessed using an auditory temporal bisection paradigm, in which the task was to compare sound stimulus durations with learned (2 and 5 s) reference intervals. We varied sound emotional valence and semantic identity (behavioural salience) to create four stimulus conditions: pleasant environmental (running water), unpleasant environmental (machine noise), pleasant human (laughter) and unpleasant human (crying) sounds. Psychometric functions were constructed to assess sound duration estimation (bisection point) and sensitivity (Weber’s ratio), and participant groups were compared using linear mixed regression models. Neuroanatomical associations of altered subjective time perception (sound duration estimation) were assessed using voxel-based morphometry of patients’ brain MRI images. All participants perceived environmental sounds as lasting longer than human sounds, unpleasant environmental sounds as longer than pleasant environmental sounds and pleasant human sounds as longer than unpleasant human sounds (all *P* < 0.05). In dementia syndromes, the effect of sound semantic category was accentuated: patients with nonfluent variant primary progressive aphasia overestimated environmental sound duration, while patients with logopenic aphasia underestimated the duration of human sounds, relative to controls (*P* < 0.05). In addition, patients with typical Alzheimer’s disease and behavioural variant frontotemporal dementia discriminated sound duration changes less sensitively than controls, while patients with semantic variant primary progressive aphasia discriminated sound duration more sensitively than other syndromic groups (*P* < 0.05). Neuroanatomical correlates of auditory duration perception were identified for different sound categories, in distributed cortical areas previously implicated in the pathogenesis of these diseases (all significant at *P* < 0.05, after correction for multiple voxel-wise comparisons in pre-specified regions of interest): precuneus (environmental sounds), supramarginal gyrus (pleasant human sounds) and insula (unpleasant human sounds). Our findings show that canonical dementia syndromes have clinical and neuroanatomical signatures of altered subjective time perception, linked to clinically relevant properties of sensory stimuli and the core pathophysiology of frontotemporal dementia and Alzheimer’s disease. The findings suggest a novel paradigm for characterizing these diseases, with diagnostic and management implications.

## Teaser

Requena-Komuro *et al*. found experienced duration of everyday sounds is altered and time sensitivity is reduced in people with different dementias compared to older controls. Time awareness signatures in dementia reflect brain atrophy profiles. This work highlights a core human experience that is vulnerable to dementia.

## Graphical Abstract

**Graphical Abstract:**
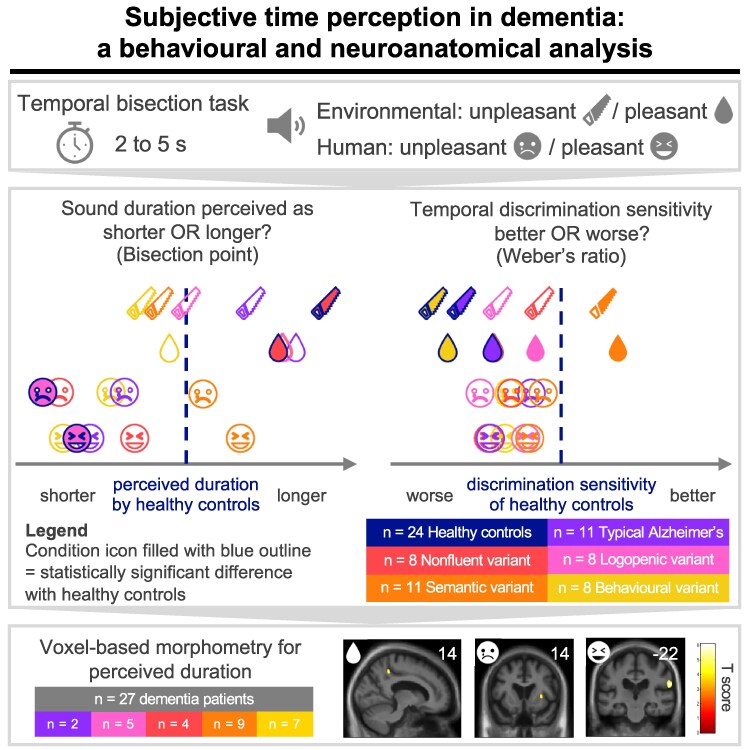

## Introduction

As philosophers and poets recognized long ago, one’s sense of elapsed time depends crucially on the psychological state and circumstances of the perceiver.^1,2^ This elasticity or modulation of subjective time is fundamental both to our awareness of the world and our experience of an embodied self^3-6^ and can be quantified psychologically.^7^ People living with dementias such as Alzheimer’s disease (AD) and frontotemporal dementia (FTD) often exhibit clinically relevant symptoms of altered temporal awareness.^8^ However, the impact of these diseases on subjective time perception remains poorly understood.

Patients with both typical amnestic and language-led (logopenic) AD show impaired awareness of extended temporal intervals encompassing autobiographical events.^9-11^ Over shorter timescales, patients with AD are inaccurate and imprecise when prospectively estimating the duration of intervals lasting several seconds.^12,13^ Alterations in subjective time estimation may manifest early in the course of AD.^14^ In the FTD spectrum, a rich diversity of phenomena linked to abnormal temporal perception and awareness has been reported, likely reflecting the clinical and pathological heterogeneity of these diseases.^15,16^ Among the major canonical variant syndromes of FTD, the behavioural variant (bvFTD) is associated with impaired processing of temporal intervals on the order of seconds,^17,18^ while patients with bvFTD and the semantic variant of primary progressive aphasia (svPPA) are prone to clockwatching and temporal rigidity, which may reflect abnormal valuation of time.^9,10,19^ These abnormalities of temporal awareness in AD and FTD arise in the context of a more general impairment of sensory change detection^20-22^ and impaired coding of the identity, behavioural salience and affective tone of sensory objects and interoceptive signals.^23-25^ Although cognitive and physiological dysfunction underpinning impaired hedonic and emotional processing is generally most prominent and widely documented in FTD syndromes,^26-31^ abnormalities of affective and emotional processing are increasingly recognized in AD.^32-35^

In the healthy brain, time perception is importantly modulated by diverse, non-temporal factors.^7,36,37^ These include affective tone (human emotional facial expressions^38-41^ and vocalizations)^42,43^, generic affective visual stimuli,^44-46^ homeostatic state,^37^ behavioural salience,^47,48^ perceptual features^49,50^ and acoustic temporal structure.^51,52^ This modulation of subjective time perception by sensory stimulus properties and homeostatic state can be interpreted neurobiologically in terms of an internal clock model.^53,54^ Key to the model is the modulation of subjective time by complex and interacting effects of emotional arousal and attention, acting to ‘gate’ a putative neural pulse generator or pacemaker. Arousal tends to open the gate, allowing more pulses and longer perceived duration, while increasing attentional demands (where these divert attentional resources away from timekeeping) lead to fewer pulses and short perceived duration.^55-58^

The neural networks that support this clock mechanism in the healthy brain closely overlap those mediating emotional and interoceptive signal processing, magnitude estimation, working memory and attention.^4,59-62^ The neuroanatomy of temporal processing is complex^61-67^: metanalyses have delineated separable substrates for processing sub-second intervals (particularly subcortical circuitry, including basal ganglia and cerebellum) and supra-second intervals (a cortical network encompassing lateral prefrontal and supplementary motor, insular and parietal areas)^61,65,67^ and temporal versus spatial intervals.^66^ Durational representations are differentially embodied, sensorimotor experience particularly engaging prefrontal areas and interoceptive experience the insula.^62,63^ The engagement of timing networks is further modulated by task, particularly the requirement for motor coding of interval duration. Furthermore, the neural coding of temporal duration is differentiated according to stimulus characteristics^62,68^ and is likely to depend fundamentally on tracking of perceptual features in the incoming sensory stream.^69^ The distributed neural networks implicated in duration perception (in particular prefrontal, insular and inferior parietal cortices) are targeted early and selectively in AD and FTD.^15,70-73^ It is therefore neurobiologically plausible that subjective time perception should be altered in FTD and AD and, further, that this alteration should be influenced by the perceptual and semantic characteristics, affective tone and salience of incoming sensory stimuli.

Here we investigated subjective temporal perception of supra-second durations and its modulation by the emotional tone and behavioural salience of sensory stimuli in a cohort of patients representing AD and all major syndromes of FTD, in relation to healthy older individuals. We used sound as the sensory vehicle: sounds always unfold in time, and the processing of sounds is essentially dynamic and entails high temporal precision.^74-76^ Moreover, deficits of duration estimation may be more evident in the auditory than the visual modality^7,18^ and may be amplified by auditory cortical dysfunction.^77-79^ We manipulated two key auditory attributes—affective valence (pleasantness) and source identity or semantic category (human vocalizations versus environmental sounds)—orthogonally in auditory stimuli of variable duration. In manipulating sound identity independently of affective valence, we sought to determine the role of behavioural salience in modulating temporal perception. Human vocalizations are both highly behaviourally salient and embodied, characteristics which are likely to modify the dynamic subjective coding of these human sounds relative to other sound categories.^80,81^ To index temporal duration perception, we adapted a widely used paradigm: the temporal bisection task.^82,83^ In this paradigm, subjects first learn to recognize the duration of a short and a long reference stimulus and are then required to determine whether each of a set of varying comparison intervals is closer to the short or the long reference duration. We assessed neuroanatomical associations of temporal duration estimation using voxel-based morphometry of patients’ brain MRI images.

Based on available evidence in the healthy brain,^38-41,43^ we hypothesized that cognitively well older individuals would overestimate the duration of unpleasant sounds compared to pleasant ones and that their duration estimates would additionally be modulated by the behavioural salience of the sounds (human vocalizations versus environmental sounds).^47,68^ Based on known profiles of perceptual, cognitive, emotional and homeostatic impairment in AD and FTD syndromes,^9,10,18,22,24,26,27,29,32^ we hypothesized that these syndromes would show differential modulation of subjective time perception relative to cognitively well older individuals: whereas AD and bvFTD would be associated with impaired sensitivity to duration change, temporal change sensitivity would be preserved in svPPA, while syndromes associated with more marked auditory perceptual dysfunction (nfvPPA and lvPPA) would show more pronounced disturbances of sound duration estimation. We additionally hypothesized that temporal processing of different stimulus categories in our neurodegenerative disease cohort would have separable neuroanatomical substrates in the right-lateralized, distributed cortical networks previously implicated in temporal perception, in particular prefrontal, parietal and insular cortices.^61-67,84-90^ Our neuroanatomical focus here was on areas previously implicated in perceptual coding of supra-second durations, as these intersect the atrophy profiles canonically associated with FTD and AD.

## Materials and methods

### Participants

Sixty consecutive patients [mean age 68.8 (range 48–77 years); 28% female] were recruited via the specialist cognitive disorders clinic of the National Hospital for Neurology and Neurosurgery and the Dementia Research Centre research participant database from September 2019 to December 2021. Fifteen patients had a diagnosis of typical memory-led AD (henceforth designated AD; 10 took part remotely due to the COVID-19 pandemic), 15 (four remote) had bvFTD, 11 (four remote) had svPPA, 10 (six remote) had nfvPPA, and nine (seven remote) had lvPPA. No formal sample size was calculated, as prior effect sizes here were unknown. All patients fulfilled consensus criteria for the relevant syndromic diagnosis,^91-93^ of mild to moderate severity. Brain MRI was consistent with the syndromic diagnosis in all patients, without evidence of significant cerebrovascular burden. Seven AD patients had CSF biomarker support for underlying AD pathology, based on local CSF phosphorylated-tau or tau/beta-amyloid reference ranges. Thirteen AD patients were taking an acetylcholinesterase inhibitor and/or memantine (Table 1). Twenty-four healthy age-matched individuals with no history of neurological or psychiatric illness were recruited via the departmental research database, 11 of whom took part remotely. None had a history of clinically significant peripheral hearing impairment. Clinical, demographic and general neuropsychological characteristics of the participant cohort are summarized in Table 1.

**Table 1:** Demographic, clinical and general neuropsychological characteristics of participant groups

Characteristic	Controls	AD	lvPPA	nfvPPA	svPPA	bvFTD
Demographic and clinical						
*N* (M/F)	12/12	7/4	6/2	5/3	7/4	5/3
Age (y)	69.4 (6.5)	71.3 (5.4)	70.4 (5.4)	69.4 (5.7)	66.0 (9.3)	67.9 (7.2)
Handedness (R/L/A)	20/3/1	11/0/0	8/0/0	8/0/0	9/1/1	8/0/0
Education (y)	16.1 (2.6)	14.4 (3.7)	15.8 (3.0)	14.3 (3.1)	15.5 (1.8)	13.6 (2.0)
Symptom duration (y)	N/A	6.3 (3.3)	5.8 (5.3)	3.1 (1.1)	5.5 (3.1)	13.0 (9.3)
T-MMSE (/27)	26.0 (1.3)*^n^*^−2^	**18.7 (3.8)**	**17.5 (6.2)**	23.4 (2.9)	**21.8 (3.9)*^n^*^−1^**	**20.9 (5.4)**
AD biomarkers^a^	N/A	6	1	N/A	N/A	N/A
AD therapies^b^	N/A	8	5	N/A	N/A	N/A
General neuropsychological assessment						
General intellect						
Performance IQ	123.6 (11.1)*^n^*^−10^	85.0 (1.4)*^n^*^−9^	101.3 (35.4)*^n^*^−5^	94.0 (21.7)*^n^*^−5^	114.3 (21.8)*^n^*^−5^	100.1 (24.4)*^n^*^−1^
Verbal IQ	123.3 (8.4)*^n^*^−10^	95.0 (14.1)*^n^*^−9^	**55.0 (0.0)*^n^*^−6^**	*74.0^n−7^	**74.8 (21.0)*^n^*^−5^**	**81.2 (24.0)*^n^*^−2^**
NART	41.5 (6.3)	32.7 (10.8)*^n^*^−1^	28.4 (14.1)	28.7 (13.8)*^n^*^−1^	**17.8 (14.5)*^n^*^−2^**	30.6 (17.4)
Executive function						
WASI Block Design (/71)	47.1 (12.5)*^n^*^−10^	5.0 (1.4)*^n^*^−9^	22.3 (31.8)*^n^*^−5^	21.0 (11.5)*^n^*^−5^	40.3 (19.9)*^n^*^−5^	22.1 (19.7)*^n^*^−1^
WASI Matrices (/32)	26.1 (3.0)	**12.4 (9.6)*^n^*^−2^**	**17.1 (8.2)**	22.0 (7.2)	22.5 (6.8)	17.3 (9.1)
Letter fluency	18.8 (4.7)	**9.9 (6.8)*^n^*^−2^**	**8.1 (5.2)**	**9.3 (8.4)*^n^*^−1^**	**9.1 (6.5)*^n^*^−2^**	**7.9 (6.8)**
Category fluency	24.6 (4.9)	**10.6 (6.4)*^n^*^−2^**	**10.1 (7.1)*^n^*^−1^**	18.3 (9.4)*^n^*^−1^	**7.7 (6.3)*^n^*^−2^**	**11.3 (6.8)**
TMT A (s)	31.1 (10.2)*^n^*^−10^	68.0 (9.9)*^n^*^−9^	63.7 (37.4)*^n^*^−5^	40.5 (7.8)*^n^*^−6^	61.0 (33.3)*^n^*^−6^	62.4 (41.1)*^n^*^−1^
TMT B (s)	60.4 (20.3)*^n^*^−10^	253.0 (66.5)*^n^*^−9^	183.0 (165.5)*^n^*^−6^	129.0 (80.6)*^n^*^−6^	148.8 (101.4)*^n^*^−6^	162.7 (109.6)*^n^*^−1^
D-KEFS Stroop: Colour (s)	30.9 (5.9)	56.5 (20.0)*^n^*^−5^	**71.3 (25.4)*^n^*^−1^**	**76.2 (37.6)**	48.8 (21.7)*^n^*^−3^	52.8 (21.6)
Word (s)	23.0 (4.0)	39.4 (16.9)*^n^*^−5^	**43.6 (13.6)*^n^*^−1^**	**57.8 (18.8)^sv; bv^**	29.4 (11.8)^nf; *n*−3^	32.7 (16.5)^nf^
Interference (s)	56.2 (11.5)	**118.4 (45.9)*^n^*^−5^**	**157.0 (73.3)*^n^*^−2^**	**112.9 (34.9)**	94.0 (39.6)*^n^*^−3^	**115.8 (56.7)**
Working memory						
DS-F (max)	6.8 (0.9)	5.2 (1.5)*^n^*^−1^	**4.5 (1.2)**	5.1 (1.9)	6.5 (0.9)	5.8 (2.3)
DS-R (max)	5.6 (1.2)	**3.3 (1.2)*^n^*^−1^**	**3.4 (1.4)**	3.9 (1.5)	4.9 (1.4)	4.1 (2.0)
Episodic memory						
RMT words (/50)	48.1 (2.5)*^n^*^−10^	39.5 (2.1)*^n^*^−9^	**31.0 (10.4)*^n^*^−5^**	*46.0^n−7^	**35.2 (8.3)*^n^*^−6^**	40.1 (10.1)*^n^*^−1^
RMT faces (/50)	40.7 (4.7)*^n^*^−10^	28.0 (4.2)*^n^*^−9^	**25.7 (1.2)*^n^*^−5^**	*34.0^n−7^	31.3 (3.7)*^n^*^−5^	34.3 (7.6)*^n^*^−2^
RMT faces short (/25)	23.4 (2.5)*^n^*^−14^	**15.9 (3.4)^nf^**	21.6 (3.8)*^n^*^−3^	22.8 (3.5)^ad; *n*−2^	19.3 (3.7)*^n^*^−7^	23.0 (1.4)*^n^*^−6^
Camden PAL (/24)	21.1 (3.6)*^n^*^−10^	5.5 (2.1)*^n^*^−9^	5.3 (9.8)*^n^*^−4^	16.7 (5.8)*^n^*^−5^	8.8 (8.8)*^n^*^−6^	**8.3 (9.1)**
Language						
GNT (/30)	25.7 (2.3)*^n^*^−1^	**12.5 (6.7)**	**8.1 (8.0)**	16.3 (9.9)	**1.5 (4.5)**	15.1 (9.6)
BPVS (/150)	147.9 (2.0)*^n^*^−1^	**136.7 (18.8)**	133.0 (23.8)	137.8 (15.6)	**86.4 (51.6)*^n^*^−2^**	122.5 (50.8)
Arithmetic						
GDA (/24)	15.6 (5.0)*^n^*^−1^	**5.0 (5.1)*^n^*^−2^**	**4.8 (5.6)*^n^*^−2^**	**6.5 (5.8)**	10.1 (6.8)*^n^*^−2^	11.0 (8.5)
Visuospatial						
VOSP (/20)	18.7 (1.7)	**14.3 (3.4)*^n^*^−1^**	**14.8 (2.9)**	17.6 (1.9)	15.6 (4.0)*^n^*^−2^	17.6 (1.5)

Mean (standard deviation) scores are shown unless otherwise indicated (standard deviations not shown when sample size equals 1). Maximum scores are shown in parentheses after tests where relevant. Patient data correspond to the number of patients who passed the practice phase of the temporal bisection task (14 patients in total failed the practice phase; see more details in text under Materials and Methods). The MMSE was administered in-person for half of the participant sample (*n* = 35) and the T-MMSE remotely for the other half; for uniformity, all MMSE scores were converted into T-MMSE scores. A reduced number of participants took part in certain tests; missing data are coded as *^n^*^−*x*^, where *x* is the number of ‘missing’ participants. Statistically significant differences between patient groups and healthy controls (*P* < 0.05) are indicated in bold. Between patient group differences are indicated as follows: ^ad^significantly different from AD; ^nf^significantly different from nfvPPA group; ^sv^significantly different from svPPA group; ^bv^significantly different from bvFTD group. *Performance from only one participant was available and was therefore compared to healthy controls group using a one sample *t*-test. ^a^ Total number of AD and lvPPA patients with positive AD biomarkers based on local CSF phosphorylated-tau or tau/beta-amyloid reference ranges. ^b^ Total number of AD and lvPPA patients who were receiving AD symptomatic therapies at the time of testing (donepezil and memantine combined: two AD, one lvPPA; donepezil only, four AD and three lvPPA; memantine only, two AD; rivastigmine only, one lvPPA). A, ambidextrous; AD, patient group with typical Alzheimer’s disease; bvFTD, patient group with behavioural variant frontotemporal dementia; BPVS, British Picture Vocabulary Scale^154^; Controls, healthy control group; D-KEFS, Delis Kaplan Executive System^155^; DS-F and DS-R, Digit Span Forward and Reverse^156^; F, female; GDA, Graded Difficulty Arithmetic test^157^; GNT, Graded Naming Test^158^; IQ, intelligence quotient; L, left-handed; lvPPA, patient group with logopenic variant primary progressive aphasia; M, male; N, number of participants; N/A, not available; NART, National Adult Reading Test^159^; nfvPPA, the patient group with nonfluent/agrammatic primary progressive aphasia; PAL, paired associates learning^160^; R, right-handed; RMT, recognition memory test^161^; svPPA, the patient group with semantic variant primary progressive aphasia; T-MMSE, telephone mini-mental state examination^162^; TMT, Trail Making Test^163^; VOSP, visual object space perception task^164^; WASI, Wechsler Abbreviated Scale of Intelligence^165^; y, years.

All participants gave informed consent. Ethical approval was granted by the University College London and National Hospital for Neurology and Neurosurgery Joint Research Ethics Committees in accordance with Declaration of Helsinki.

### Presentation of experiments

For face-to-face testing, stimuli were presented using the Eyelink Experiment Builder software (SR Research, Ottawa, Canada). Participants listened to stimuli over ATH-M50X Audio Technica® headphones through a laptop (MacBook Air, 13 inch) at a comfortable listening level (at least 70 dB). Remote testing was carried out following a procedure described previously.^94^

### Experimental stimuli

Recordings of environmental sounds and emotional vocalizations were obtained online (freesound.org, soundsnap.com) or from the Speech Communications Lab at University College London.^95^ The raw sound recordings were edited as wave files in Audacity (v2.3.3) to extract segments of duration 2, 2.5, 3, 3.5, 4, 4.5 or 5 s. Stimuli were sampled at 44.1 kHz, windowed to remove click onset/offset artefacts and fixed for root-mean-square intensity.

The auditory stimuli were chosen to represent four different sound category–valence combinations (i.e. four experimental conditions): pleasant environmental noises (brook, river); unpleasant environmental noises (angle grinder, car horn); pleasant human vocal sounds (female laughter, male laughter); and unpleasant human vocal sounds (female crying, male crying). Stimulus examples are in Supplementary material (see Table S1).

This design employed sounds whose pleasantness had previously been validated^35^ while at the same time allowing for acoustic variation across the stimulus set, to reduce the potential for idiosyncratic stimulus effects. However, for all participants, we assessed whether their individual valence ratings were in line with those predicted for each sound category, as follows. Thirty-six stimuli from the main experiment representing the four sound conditions (each with duration 3.5 s) were administered in a randomized order; the task on each trial was to rate the pleasantness of the sound, using a mouse click on a sliding scale bounded by ‘extremely unpleasant’ and ‘extremely pleasant’ (accompanied by matching facial expression icons). The mouse position was automatically converted into a score between 0 (‘extremely unpleasant’) and 100 (‘extremely pleasant’) for further analysis. For each sound, a mean valence rating score of 50 or higher was considered as pleasant and a score below 50 as unpleasant.

Most participants gave sound valence ratings that matched the pre-designated valence categories, albeit with wide individual variation within categories (see Figs S1 and S2 for detailed analysis); for seven healthy control participants, the mean valence rating of at least one sound condition did not fit the pre-defined valence category and the corresponding sounds were therefore reassigned to the appropriate individual valence category before analysis. A secondary analysis was conducted incorporating patients’ own valence ratings, to assess the impact of deviations from pre-assigned valence categories (details in Supplementary material, specifically Tables S2 and S3).

### Temporal bisection task

The temporal bisection task is a classical psychological paradigm typically used to evaluate the influence of emotion on the perception of short time intervals. On each trial, participants are asked to attend to a stimulus (here a sound) of varying duration and then to categorize each stimulus as a ‘short’ interval or a ‘long’ interval by responding with a keypress. In line with previously published protocols,^43,45,96^ here we assessed seven different durations within a range of 2–5 s (2, 2.5, 3, 3.5, 4, 4.5, 5 s) pertinent to subjective experience of short time intervals in daily life.

Following the non-partition method, the experiment was divided into a training phase and an experimental phase. Participants were first familiarized with the experimental paradigm using four practice trials with sounds (gibbon call, rain) not reused subsequently, to ensure they understood the task. The training phase comprised 16 trials in which participants learned the duration of the short (2 s) and long (5 s) duration references for the eight sounds used subsequently in the experiment; on each trial, the task was to decide whether the sound was ‘short’ or ‘long’, with a verbal response. Visual and verbal feedback about the participant’s performance was given on every training trial. A score >80% (13/16) trials correct was required to enter the experimental phase. Participants were allowed to repeat the training trials (with the same sounds) if they failed initially but were excluded from the experimental phase if they failed a second time. Fourteen patients (seven bvFTD, two nfvPPA, one lvPPA, four AD) failed the training phase and were therefore removed from the final analysis cohort (details in Supplementary material text and Table S4). A flow diagram detailing the number of patients included at each stage of the study is presented in Fig. S3.

In the experimental phase, participants were presented with sound stimuli representing the five intermediate durations (2.5, 3, 3.5, 4, 4.5 s) as well as the two reference durations (2 and 5 s). The task on each trial was to categorize the duration of the sound as closer to either the short or the long reference durations, and participants provided their answers in the same way as for the training trials. Here, there were a total of 224 experimental trials, which were divided into eight blocks. Each experimental block represented all 28 (7 × 4) sound duration—condition combinations, which were presented in a pseudo-randomized order (such that no two sounds from the same sound condition were presented consecutively) and with a varying inter-trial interval (between one and three seconds) to reduce expectancy effects. The order of the blocks was further randomized across participants. Participants were allowed to take short breaks between blocks. Importantly, participants were not told that the temporal bisection task examined the effect of emotion on time perception prior to taking part in the experiment.

### Control tasks and questionnaires

Two additional control tasks were administered after the temporal bisection task to evaluate the influence of potential confounding factors. These comprised a pure tone duration categorization task, to assess participants’ ability to make basic temporal decisions about auditory stimuli, and an auditory recognition task based on sound-picture matching, to assess semantic knowledge of the sounds used in the main experiment. Details of these tests are described in Supplementary material text and Fig. S4. The percentage of trials with correct responses on each of these two tests was calculated for all participants.

Participants also completed musical experience^97^ and mood^98^ questionnaires. When assessed face-to-face, they also underwent pure tone audiometry following a standard clinical protocol (details in Supplementary material).

### Analysis of clinical and behavioural data

All statistical analysis of behavioural data was performed using Stata (StataCorp, College Station, TX, USA).

For the analysis of continuous variables, a one-way ANOVA was used if assumptions of homoscedasticity and normality, verified using Levene’s test and Q–Q plot of residuals, were satisfied. If assumptions were not met, the non-parametric equivalents (Mann–Whitney rank-sum test and Kruskal–Wallis *H* test, respectively) were performed instead. For comparison of categorical variables (e.g. sex, handedness), a chi-square test was used, unless the expected counts were small, in which case a Fisher’s exact test was used instead. A statistical threshold of *P* < 0.05 was accepted for all tests.

For each participant who completed the experimental phase of the temporal bisection task, the proportion of long responses was computed by dividing the number of long responses by the number of blocks for each sound condition at each duration. The probability of a ‘long’ response for each sound condition was then plotted as a function of duration. A psychometric curve was then estimated for each condition using Psignifit (version 4.0; https://uni-tuebingen.de/en/fakultaeten/mathematisch-naturwissenschaftliche-fakultaet/fachbereiche/informatik/lehrstuehle/neuronale-informationsverarbeitung/research/software/psignifit/), a MATLAB-operated toolbox.^99^ Following previous analysis methods,^40,45,96^ temporal bisection point and Weber’s ratio were extracted from each psychometric curve for further statistical analyses. The temporal bisection point corresponds to the stimulus duration at which the curve reaches 50% probability of a ‘long’ response; a lower bisection point would reflect overestimation of the corresponding sound category duration relative to others. Weber’s ratio or the coefficient of variation was calculated as follows: [(duration at P_long_ = 0.75) − (duration at P_long_ = 0.25)]/bisection point. Weber’s ratio gives an indication of the discrimination sensitivity, i.e. a higher value, corresponding to a shallower slope, would indicate a lower discrimination sensitivity. These parameters were averaged across participants from the same group. Data collected remotely were merged with data collected face-to-face; we have previously shown that a remote testing setting does not degrade auditor test performance in this cohort.^94,100^

The following variables were considered as potential confounders on temporal bisection performance: mood score before completing the temporal bisection task, hearing score, past musical experience and scores on the temporal bisection training phase, sound recognition and pure tone duration categorization control tasks. To determine whether to include these as nuisance covariates, we assessed whether participant group mean scores for each candidate confounding variable differed significantly in one-way ANOVAs.

To assess experimental effects, a linear mixed model incorporating diagnostic group, semantic category and valence as fixed effects and participant identity as the only random effect was computed for bisection point and Weber’s ratio separately. For Weber’s ratio, the assumption of normality of residuals was violated, and a logarithmic transformation was therefore applied. For both parameters, there was no violation of homoscedasticity nor significant outliers or multicollinearity, and further statistical testing confirmed the models were correctly specified. When an effect was found, *post hoc* group comparisons were performed.

In addition, individual patient bisection point and Weber’s ratio values were averaged across the four sound conditions to conduct exploratory correlation analyses using Pearson’s or Spearman’s tests. Correlations were assessed between temporal bisection task performance parameters and forward and reverse digit span (indexing auditory working memory), WASI matrices score (indexing nonverbal executive function) and mini-mental state examination (MMSE) score (indexing overall cognitive impairment).

### Brain image acquisition and analysis

For all patients, volumetric T1 MR brain images were acquired on a Siemens Prisma 3T MRI scanner and pre-processed following standard protocols (see Supplementary material).

Across the combined patient cohort, we ran a full factorial model to assess associations of regional grey matter volume with duration estimation differences between controls and patients for each sound condition (environmental unpleasant, environmental pleasant, human unpleasant, human pleasant). Specifically, the model incorporated the signed difference calculated as the bisection point averaged across all control participants for a particular sound condition minus the patient’s individual bisection point for that same sound condition. The diagnostic group (as a five-level factor) and three nuisance covariates (age, total intracranial volume, MMSE score) were included as covariates. For every sound condition, we assessed associations over the combined patient cohort between regional grey matter volume and signed differences in patient mean temporal bisection point subtracted from the healthy control group mean bisection point. If the overall sign of these (disease-associated) differences is positive, an inverse (negative) correlation would indicate regional grey matter atrophy; whereas if the overall sign of the differences is negative, a direct (positive) correlation would indicate atrophy.

Statistical parametric maps were generated using an initial cluster-forming threshold *P* < 0.001 and evaluated at peak voxel statistical significance level *P* < 0.05, after family-wise error (FWE) correction for multiple voxel-wise comparisons, separately within individual pre-specified neuroanatomical regions of interest. Selection of these regions was informed by previous studies on the neuroanatomy of interval timing in the healthy brain. Regions comprised the dorsolateral prefrontal cortex,^86^ insula and frontal operculum,^66,89,90^ inferior parietal lobule,^84,87^ supplementary motor area^61^ and precuneus.^85,88^ They were defined for the right hemisphere only using the Harvard-Oxford Brain Atlas (http://fsl.fmrib.ox.ax.uk/fsl/fslwiki/Atlases; see Fig. S5 in Supplementary material online), in line with previous evidence for preponderantly right-sided (or bilateral) neuroanatomical correlates of time perception in previous studies^64,66,84-87,90^ and to avoid severe disease-related atrophy distortions in the left hemisphere.

## Results

### General participant characteristics and control task performance

Participant groups did not significantly differ in age, education, sex or handedness distributions, and syndromic groups did not differ in mean symptom duration (all *P* > 0.05, Table 1). The AD, lvPPA, svPPA and bvFTD groups all scored worse on the MMSE than healthy controls (Kruskal–Wallis, *X*^2^ = 33.981, *P* < 0.001).

Mean valence ratings across all participants matched pre-defined categories, and the unpleasant/pleasant ratings were similar between semantic categories (see Supplementary material text and Figs S1 and S2). Mean valence ratings (M) and corresponding standard deviation (SD) for each sound condition were as follows: environmental unpleasant, M = 26, SD = 20; environmental pleasant, M = 74, SD = 16; human unpleasant, M = 25, SD = 18; and human pleasant, M = 68, SD = 18. In addition, 22 patients (five AD, four nfvPPA, eight svPPA, five bvFTD) gave sound valence ratings that did not fit pre-defined categories (see Table S2). Results from the secondary analysis considering these deviations are presented in Table S3 in Supplementary material online and were broadly similar to the results from the main analysis presented below.

There were no statistically significant between-group differences in hearing threshold, mood score, musical background nor percentage correct scores on the tone duration control task, sound recognition task or the training phase of the temporal bisection task (all *P* > 0.05; Table 2). None of these measures was therefore included as nuisance covariates in the linear mixed models presented below.

**Table 2:** Group mean performance profiles on questionnaires and experimental control tasks

Diagnosis	Controls	AD	lvPPA	nfvPPA	svPPA	bvFTD
N participants	24	11	8	8	11	8
Hearing threshold	26.3 (11.5) *^n^*^−14^	43.5 (3.5)*^n^*^−9^	27.7 (9.5)*^n^*^−5^	31.3 (15.0) *^n^*^−5^	25.4 (10.0) *^n^*^−6^	33.0 (7.8)*^n^*^−1^
Mood score (/20)	7.7 (2.5)*^n^*^−10^	8.8 (3.7)*^n^*^−3^	5.8 (1.8)*^n^*^−2^	6.7 (4.5)*^n^*^−2^	6.2 (2.3)*^n^*^−6^	7.0 (1.4)*^n^*^−6^
Musical background (/4)	1.4 (1.4)*^n^*^−2^	1.4 (1.0)*^n^*^−2^	2.2 (1.8)*^n^*^−2^	1.0 (1.3)*^n^*^−1^	1.8 (1.6)*^n^*^−2^	1.6 (0.9)
Pure tone duration score (%)	97.8 (4.1)	94.1 (9.7)*^n^*^−1^	95.7 (4.8)	94.4 (9.1)	98.9 (3.6)	96.9 (6.2)
Sound recognition score (%)	N/A	98.9 (3.6)	100.0 (0.0)	100.0 (0.0)	96.0 (13.3)	97.8 (4.5)
Temporal bisection training phase score (%)	94.2 (6.2)	88.0 (6.7)	91.6 (6.6)	90.9 (5.8)	94.5 (5.9)	89.0 (9.4)

Group mean (standard deviation) values are presented. Maximum scores are shown in parentheses for relevant variables. A higher mood score indicates worse mood; a high score on the musical background questionnaire indicates extensive musical experience. The pure tone duration categorization task and the sound recognition task are described in further details in the main text. The practice phase score shows the number of correct practice trials in the temporal bisection task. Participants who scored over 80% (13 correct practice trials out of 16) went on to complete the experimental phase (see text). A reduced number of participants completed certain tasks (audiometry was only performed with participants tested face-to-face; the mood questionnaire was only administered to the remote cohort; other missing datapoints due to limited testing time). Missing data are coded as *^n^*^−*x*^, where *x* is the number of ‘missing’ participants. There were no statistically significant differences between participant group means on any of the variables listed here. AD, the patient group with typical Alzheimer’s disease; bvFTD, the patient group with behavioural variant frontotemporal dementia; controls, the healthy control group; lvPPA, the patient group with logopenic variant primary progressive aphasia; N/A, not administered; nfvPPA, the patient group with nonfluent/agrammatic primary progressive aphasia; svPPA, the patient group with semantic variant primary progressive aphasia.

### Bisection point (perceived sound duration)

**Figure 1:**
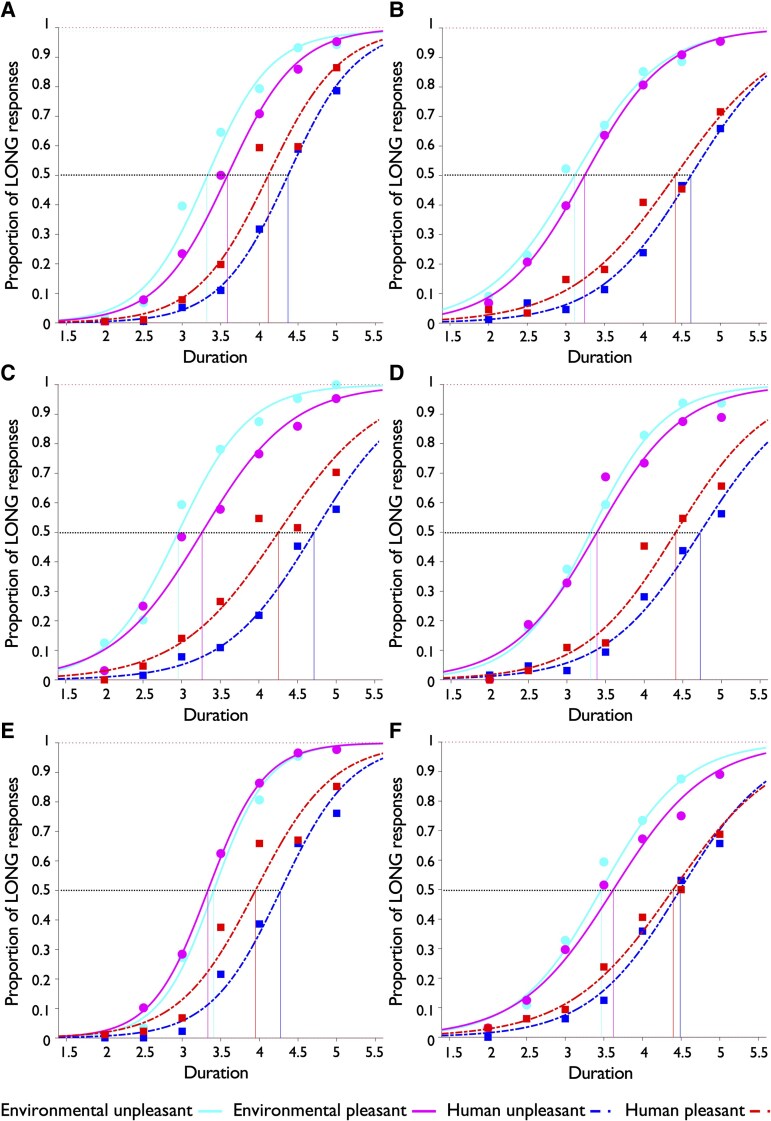
**Mean psychometric curves for each sound condition per each participant group.** The number of long responses and the total number of trials available at each stimulus duration and for each sound condition have been averaged across participants (*n* = 24 CTL and *n* = 46 patients) from the same group and used to estimate psychometric curves for that group (A, healthy controls; B, AD; C, nfvPPA; D, lvPPA; E, svPPA; F, bvFTD). Environmental sound curves are shown in solid lines and corresponding datapoints using round circles, while human sound curves are shown in dash-dotted lines and corresponding datapoints using squares. A different colour is used for each curve as indicated in legend (environmental unpleasant, cyan; environmental pleasant, magenta; human unpleasant, blue; human unpleasant, red). The horizontal black-dotted line at y = 0.5 represents an equal proportion of short and long responses and crosses each psychometric curve at its corresponding bisection point which can be read on the *x*-axis. The top horizontal dotted line represents the upper asymptote, i.e. the probability of responding long at infinitely long durations. A curve that is further to the right indicates underestimation of the corresponding duration by participants, while a curve further to the left indicates overestimation. Linear mixed models were performed to model bisection point and Weber’s ratio parameters separately (see Materials and Methods for more details). The linear mixed model corresponding to the bisection point revealed a main effect of semantic category, and significant interactions between semantic category and diagnosis and semantic category and valence (all *P* < 0.001). The linear mixed model corresponding to the Weber’s ratio revealed main effects of valence category (*P* < 0.001) and diagnosis (*P* = 0.021) and a significant interaction between semantic category and diagnosis (*P* = 0.043). Full statistical output is described in main text and in Table 3. AD, patient group with typical Alzheimer’s disease; bvFTD, patient group with behavioural variant frontotemporal dementia; lvPPA, patient group with logopenic variant primary progressive aphasia; nfvPPA, patient group with nonfluent/agrammatic primary progressive aphasia; svPPA, patient group with semantic variant primary progressive aphasia.

**Figure 2:**
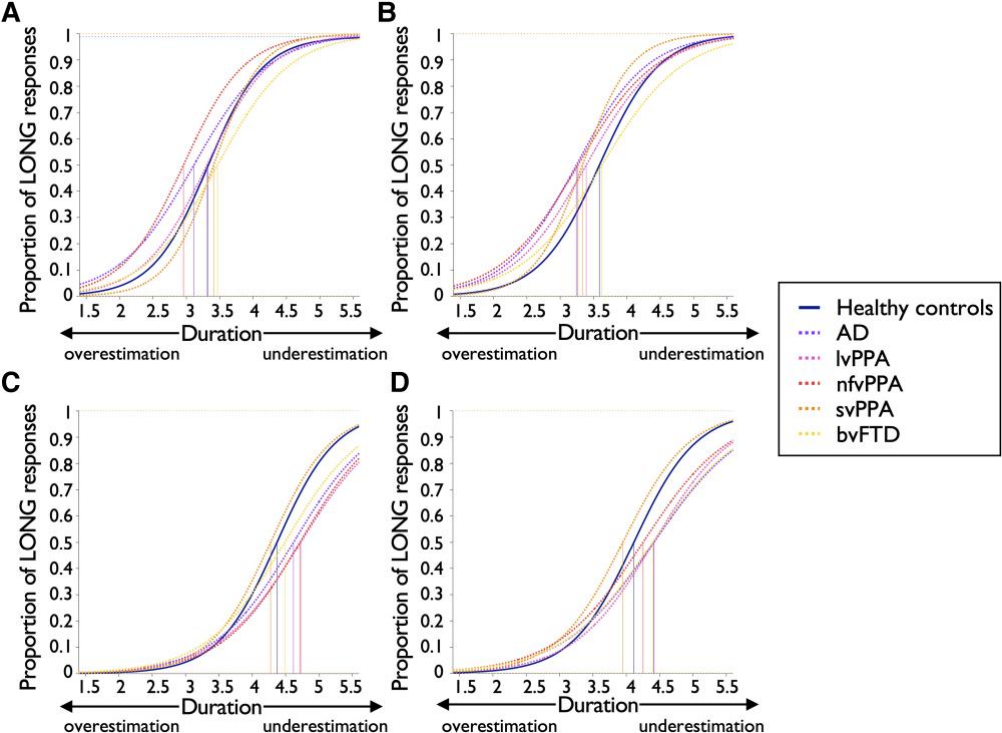
**Mean psychometric curves for each participant group per sound condition.** The number of long responses and the total number of trials available at each stimulus duration and for each sound condition (**A**, environmental unpleasant; **B**, environmental pleasant; **C**, human unpleasant; **D**, human pleasant) have been averaged across participants from the same group and used to estimate psychometric curves for that group (*n* = 24 CTL and *n* = 46 patients). The control group is shown in a solid line while patient groups are shown in dotted lines using the colour scale indicated in the legend. The top horizontal dotted line represents the upper asymptote, i.e. the probability of responding long at infinitely long durations. A curve that is further to the right indicates underestimation of the corresponding duration by participants, while a curve further to the left indicates overestimation. Linear mixed models were performed to model bisection point and Weber’s ratio parameters separately (see Materials and Methods for more details). The linear mixed model corresponding to the bisection point revealed a main effect of semantic category and significant interactions between semantic category and diagnosis and semantic category and valence (all *P* < 0.001). The linear mixed model corresponding to the Weber’s ratio revealed main effects of valence category (*P* < 0.001) and diagnosis (*P* = 0.021) and a significant interaction between semantic category and diagnosis (*P* = 0.043). Full statistical output is described in main text and in Table 3. AD, the patient group with typical Alzheimer’s disease; bvFTD, the patient group with behavioural variant frontotemporal dementia; lvPPA, the patient group with logopenic variant primary progressive aphasia; nfvPPA, the patient group with nonfluent/agrammatic primary progressive aphasia; svPPA, the patient group with semantic variant primary progressive aphasia.

**Figure 3:**
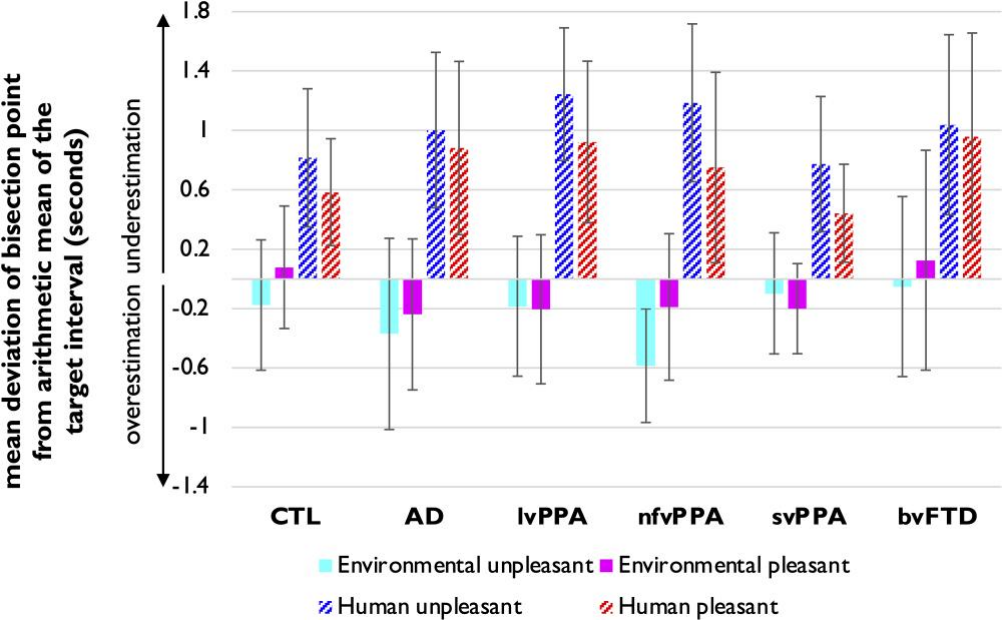
**Mean deviation of bisection point from the arithmetic mean for each sound condition and participant group.** The mean deviation of temporal bisection point (estimated sound duration) from the arithmetic mean of the target interval (3.5 s in elapsed physical clock time) has been computed for each sound condition and each participant group (*n* = 24 CTL and *n* = 46 patients). Positive deviations indicate duration underestimation, while negative deviations indicate duration overestimation. Each sound condition is displayed in a different colour as indicated in the legend (as in Fig. 1: environmental unpleasant, cyan; environmental pleasant, magenta; human unpleasant, blue; human unpleasant, red). Error bars correspond to standard deviations. AD, the patient group with typical Alzheimer’s disease; bvFTD, the patient group with behavioural variant frontotemporal dementia; CTL, healthy controls; lvPPA, the patient group with logopenic variant primary progressive aphasia; nfvPPA, the patient group with nonfluent/agrammatic primary progressive aphasia; svPPA, the patient group with semantic variant primary progressive aphasia.

Mean bisection point and Weber’s ratio values are summarized in Table 3. Psychometric curves are shown separately for each participant group in Fig. 1 and each sound condition in Fig. 2, and deviations of each participant group’s bisection point from the arithmetic mean of the target interval are shown in Fig. 3.

**Table 3:** Mean psychometric parameters on temporal bisection task for each sound condition and participant group

Diagnosis	Healthy controls		AD		lvPPA		nfvPPA		svPPA		bvFTD	
Sound condition	Env	Hum	Env	Hum	Env	Hum	Env	Hum	Env	Hum	Env	Hum
Bisection point												
Unpleasant	3.3 (0.4)	4.3 (0.5)	3.1 (0.6)	4.5 (0.5)	3.3 (0.5)	**4.7 (0.4)^sv^**	**2.9 (0.6)^bv^**	4.7 (0.6)	3.4 (0.4)	4.3 (0.5)^lv,bv^	3.4 (0.4)^nf^	4.5 (0.5)^sv^
Pleasant	3.6 (0.4)	4.1 (0.4)*^n^*^−3^	3.3 (0.5)	4.4 (0.6)	3.3 (0.5)	**4.4 (0.5)^sv^**	**3.3 (0.7)^bv^**	4.3 (0.7)	3.3 (0.3)	3.9 (0.3)^lv,bv^	3.6 (0.5)^nf^	4.5 (0.6)^sv^
Weber’s ratio												
Unpleasant	0.20 (0.07)	0.18 (0.06)	**0.27 (0.07)**	0.20 (0.12)	0.25 (0.08)	0.24 (0.08)	0.22 (0.10)	0.21 (0.08)	0.17 (0.04)^ad, lv, nfv, bv^	0.19 (0.05)	**0.29 (0.17)**	0.21 (0.04)
Pleasant	0.23 (0.08)	0.22 (0.07)*^n^*^−3^	**0.28 (0.11)**	0.27 (0.12)	0.25 (0.07)	0.24 (0.10)	0.28 (0.11)	0.27 (0.09)	0.19 (0.09)^ad, lv, nfv, bv^	0.24 (0.08)	**0.31 (0.15)**	0.26 (0.09)

Group mean (standard deviation) bisection point values (seconds) and mean (standard deviation) Weber’s ratio values are shown for the four sound conditions. A higher bisection point value indicates underestimation of the sound duration for the corresponding sound condition; a higher Weber’s ratio value indicates a lower temporal discrimination sensitivity for the corresponding sound condition. The number of missing participants as a result of sound reassignment for healthy controls is coded as *n*−*x*, where *x* is the number of ‘missing’ participants. Significant differences between healthy controls and patient groups are indicated in bold. Specifically, there was a significant interaction between semantic category and diagnosis for the bisection point analysis indicating that nfvPPA patients overestimated the duration of environmental sounds irrespective of their valence compared to healthy controls and that lvPPA patients underestimated the duration of human sounds irrespective of their valence compared to healthy controls. In addition, compared to bvFTD patients, nfvPPA patients overestimated the duration of environmental sounds, and svPPA patients overestimated the duration of human sounds, while lvPPA patients underestimated the duration of human sounds compared to svPPA patients. For the Weber’s ratio analysis, there was a significant interaction between semantic category and diagnosis indicating that both AD and bvFTD patients had lower temporal discrimination sensitivity for environmental sounds compared to healthy controls. In additions, svPPA patients had higher discrimination sensitivity for environmental sounds compared to all other patient groups. Between patient group differences are indicated as follows: ^ad^significantly different from AD; ^lv^significantly different from lvPPA; ^nf^significantly different from the nfvPPA group; ^sv^significantly different from the svPPA group; ^bv^significantly different from the bvFTD group. Additional details are given in the main text. AD, the patient group with typical Alzheimer’s disease; bvFTD, the patient group with behavioural variant frontotemporal dementia; Controls, the healthy control group; Env, environmental sound category; Hum, human sound category; lvPPA, the patient group with logopenic variant primary progressive aphasia; nfvPPA, the patient group with nonfluent/agrammatic primary progressive aphasia; svPPA, the patient group with semantic variant primary progressive aphasia.

The linear mixed model corresponding to the bisection point revealed a main effect of semantic category and significant interactions between semantic category and diagnosis and semantic category and valence (all *P* < 0.001). The main effect of semantic category indicated that across groups, participants significantly underestimated the duration of human sounds compared to environmental sounds (*z* = 21.16, *P* < 0.001). No significant three-way (diagnosis*semantic category*valence) interaction or other significant effects were identified.

*Post hoc* comparisons of the interaction between valence and semantic categories indicated that across groups, participants overestimated the duration of environmental unpleasant sounds compared to environmental pleasant sounds (*z* = 2.34, *P* = 0.019), while they underestimated the duration of human unpleasant sounds compared to human pleasant sounds (*z* = −3.69, *P* < 0.001).

*Post hoc* comparisons of the interaction between diagnosis and semantic category indicated that nfvPPA patients significantly overestimated the duration of environmental sounds compared to healthy controls (*z* = −2.03, *P* = 0.043) and lvPPA patients significantly underestimated the duration of human sounds compared to healthy controls (*z* = 2.28, *P* = 0.023). There were also statistically significant differences between patient groups: nfvPPA patients overestimated the duration of environmental sounds compared to bvFTD patients (*z* = −2.08, *P* = 0.038), lvPPA patients underestimated the duration of human sounds compared to svPPA patients (*z* = 2.50, *P* = 0.013), and svPPA patients overestimated the duration of human sounds compared to bvFTD patients (*z* = −2.04, *P* = 0.041).

For all participant groups (Fig. 3), underestimated duration of human sounds corresponded to mean positive deviation of the temporal bisection point from arithmetic mean (physical clock) duration; this effect was largest for lvPPA patients. Overestimated duration of environmental sounds corresponded to mean negative deviation from arithmetic mean for most participant groups and was largest for nfvPPA patients.

### Weber’s ratio (sensitivity of sound duration discrimination)

The linear mixed model corresponding to the Weber’s ratio revealed main effects of valence category (*P* < 0.001) and diagnosis (*P* = 0.021) and a significant interaction between semantic category and diagnosis (*P* = 0.043). No other significant interactions or main effects were identified. The main effect of valence indicated that across all participants, discrimination sensitivity was significantly higher for unpleasant sounds compared to pleasant sounds (*z* = 4.11, *P* < 0.001).

*Post hoc* comparisons for the main effect of diagnosis showed that patients with bvFTD (*z* = 2.45, *P* = 0.014) and AD (*z* = 2.07, *P* = 0.039) had lower discrimination sensitivity than healthy control participants. In addition, svPPA patients had higher discrimination sensitivity than patients with bvFTD (z = −2.63, *P* = 0.009), AD (z = 2.29, *P* = 0.022) and lvPPA (*z* = 2.01, *P* = 0.045). *Post hoc* comparisons of the interaction between diagnosis and semantic category revealed significant group differences in discrimination sensitivity for environmental sounds, but not human sounds. For environmental sounds, bvFTD (*z* = 2.38, *P* = 0.017) and AD (*z* = 2.48, *P* = 0.013) patients had lower discrimination sensitivity than healthy controls, while svPPA patients had higher discrimination sensitivity than patients with bvFTD (*z* = −3.50, *P* < 0.001), AD (*z* = 3.66, *P* < 0.001), lvPPA (*z* = 2.56, *P* = 0.010) and nfvPPA (*z* = 2.31, *P* = 0.021).

### Correlations between temporal bisection performance and general cognitive functions

Across the entire patient cohort, there were no statistically significant correlations between temporal bisection task performance measures and average hearing threshold, mood or musical background nor with performance on the temporal bisection training phase, tone duration or sound recognition tasks. Mean temporal bisection point and Weber’s ratio also did not correlate with the mean MMSE score. However, there were statistically significant inverse correlations of increasing mean bisection point value (i.e. sound duration underestimation) with forward digit span (Spearman’s rho = −0.438, *P* = 0.003) and reverse digit span (Spearman’s rho = −0.305, *P* = 0.042). There was also a statistically significant inverse correlation between mean Weber’s ratio value and WASI matrices score (Spearman’s rho = −0.313, *P* = 0.038). There were no other statistically significant correlations between neuropsychological task performance and temporal estimation parameters.

### Neuroanatomical results

**Figure 4:**
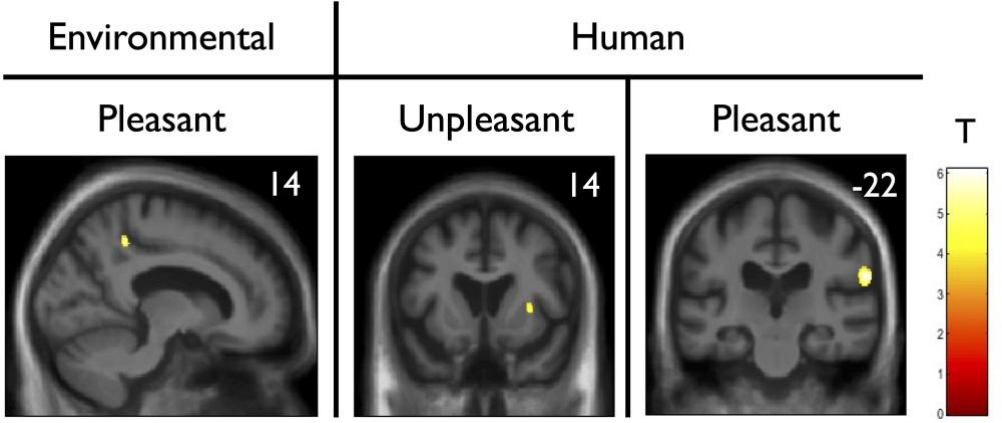
**Neuroanatomical associations of sound duration overestimation in the patient cohort.** The figure shows statistical parametric maps of regional grey matter volume inversely correlated with (i.e. grey matter atrophy correlated with) increasing differences in sound duration estimation in the combined patient cohort (*n* = 27) versus healthy older participants, in different sound conditions, obtained from voxel-based morphometry analysis (see Materials and Methods for more details and full statistical output in the main text and in Table 4). Associated grey matter regions comprised the right precuneus for environment pleasant sounds (left panel), the right insula for human unpleasant sounds (middle panel) and the right supramarginal gyrus for human pleasant sounds (right panel). For display purposes, maps are displayed on coronal sections of the group mean T_1_-weighted MRI brain image in MNI (Montreal Neurological Institute) space, thresholded at *P* < 0.001 uncorrected for multiple voxel-wise comparisons over the whole brain (areas significant at pFWE < 0.05 after correction for multiple comparisons within pre-specified neuroanatomical regions of interest are listed in Table 4). The colour bar indicates voxel-wise *T* scores (indicated as T in figure), and the plane of each section is given by the *x*-coordinate (mm) for the precuneus and the *y*-coordinate for the other two neuroanatomical regions in MNI space (top-right corner of the corresponding panel).

Significant grey matter associations of sound duration estimation differences (with respect to controls) across the entire patient cohort for each sound condition are summarized in Table 4, all thresholded at p_FWE_ < 0.05 within pre-specified anatomical regions of interest. The corresponding statistical parametric maps are shown in Fig. 4.

**Table 4:** Neuroanatomical associations of sound duration estimation differences in the patient cohort

Sound condition	Region	Side	Cluster (voxels)	Peak (mm)			T score	P_FWE_
				x	y	z		
Environmental pleasant	Precuneus	R	34	14	−44	48	5.21	0.019
Human unpleasant	Anterior insula	R	22	32	14	4	4.43	0.041
Human pleasant	Supramarginal gyrus	R	171	63	−22	24	6.1	0.005

The table presents the regions where regional grey matter volume was inversely associated with a difference in duration estimation between healthy participants and patients for the sound condition shown, over the combined patient cohort. Coordinates of local maxima are in standard MNI space. *P* values were all significant (*P* < 0.05) after family-wise error (FWE) correction for multiple voxel-wise comparisons within pre-specified anatomical regions of interest (see Fig. S5).

Based on psychometric data (Table 3; Figs 2 and 3), pathological differences in temporal bisection point versus healthy controls were defined in the patient cohort as overall negatively signed for environmental sounds and overall positively signed for human sounds. Across all patient groups, increased differences (versus the control group) in perceived duration of pleasant environmental sounds were significantly correlated with reduced grey matter in right precuneus. Across all patient groups, increased differences in perceived duration of unpleasant human sounds were significantly correlated with reduced grey matter in the right anterior insular cortex, while increased differences in perceived duration of pleasant human sounds were correlated with reduced grey matter in right supramarginal gyrus. No other neuroanatomical associations were found at the prescribed significance threshold.

## Discussion

Here, using a classic paradigm of time interval judgement with naturalistic sounds of everyday life, we have shown that subjective temporal experience in canonical syndromes of FTD and AD is differentially modulated, relative to cognitively well older individuals, by the emotional tone and semantic category (behavioural salience) of incoming sensory information. All participants perceived environmental sounds as lasting longer than human sounds, and this category effect was further modulated by valence: unpleasant environmental sounds were perceived as lasting longer than pleasant environmental sounds, whereas pleasant human sounds were perceived as lasting longer than unpleasant human sounds. All participants discriminated unpleasant sounds more sensitively than pleasant sounds. In dementia syndromes, the effect of sound semantic category was accentuated, in that patients with nfvPPA overestimated the duration of environmental sounds and patients with lvPPA underestimated the duration of human sounds, relative to cognitively well older listeners. In addition, patients with AD and bvFTD discriminated sound duration changes less sensitively than controls, and patients with svPPA discriminated sound duration more sensitively than other syndromic groups. Temporal bisection task performance correlated with neuropsychological measures pertaining to working memory and executive function. Structural neuroanatomical correlates of disease effects on auditory duration perception were identified in right-sided insular and parietal cortices.

The present findings in cognitively well older adults extend previous evidence for stimulus-induced modulation of subjective duration in young adults.^37-40^ Human vocalizations are in general more behaviourally salient than environmental noises, as they frequently demand a specific behavioural response and capture correspondingly greater, non-temporal attentional resources. Interpreted with respect to the internal clock model of subjective time perception, the duration of vocalizations (particularly if negatively valenced) would accordingly tend to be underestimated relative to environmental sounds.^42,44,45^ On the other hand, unpleasant environmental sounds are often arousing: the clock model would predict the duration of such sounds would tend to be overestimated.^39,43^ We are cautious about over-attributing the apparent effect of sound valence on the sensitivity of temporal discrimination here: the evidence that stimulus valence per se (rather than attentional or other factors) modulates the sensitivity of sensory discrimination is quite limited.^45,101,102^

The effects of neurodegenerative disease here manifested as a caricature of this basic physiological template of subjective duration estimation, the observed disease profiles aligning with previous evidence for abnormal auditory perceptual and affective auditory processing in these syndromes. Overestimation of environmental sound duration in nfvPPA may reflect an abnormal auditory arousal response to auditory stimuli and abnormal filtering of auditory stimuli irrespective of valence, in line with other work in this syndrome.^20,32,35,103,104^ Given that duration underestimation inversely correlated with auditory digit span across the entire patient cohort, underestimation of human sound duration in the lvPPA group may at least in part reflect the reduced auditory working memory capacity characteristic of this syndrome.^105^ It may also reflect paradoxically enhanced emotional and sensory salience processing,^31,106^ as well as abnormal integration of incoming vocal information with interoceptive signals during neural timekeeping.^38,107,108^ Among the syndromes studied here, nfvPPA and lvPPA have been found to have the most prominent impairments of central auditory perception.^77,109-112^ The possibility that altered modulation of perceived sound duration in these syndromes may at least in part arise from impaired encoding of acoustic spectrotemporal features. Indeed, the environmental sounds chosen here (angle grinder, car horn, brook, river) are spectrally quite uniform, uninterrupted acoustic streams, while the human sounds (crying, laughter) are complex, spectrally dynamic and anisochronously temporally segmented: these acoustic properties may have contributed to the category effect on duration estimates across the participant cohort. It has been suggested that normal listeners may perceive continuous tones as lasting longer than anisochronous sound sequences,^51^ and this effect may be more generally attributable to stimulus complexity.^113^

Our findings further suggest that the disease-associated alteration of perceived sound durations may depend on retained sensitivity for detecting changes in sound duration. Sensitivity was preserved in the nfvPPA and lvPPA groups (which showed durational perceptual modulation) but reduced in the bvFTD and typical AD groups (which did not). Reduced temporal discrimination sensitivity is likely attributable to different mechanisms in AD and bvFTD. In AD, dysfunction of cholinergic circuitry has been linked to disturbances of temporal awareness via impaired cortical short latency afferent inhibition.^10^ More specifically, cholinergic deficiency may hamper the encoding of learned durations in reference memory,^114-117^ in line with pharmacological evidence that duration discrimination sensitivity is attenuated by diminished cholinergic tone.^118^ In bvFTD, a more profound impairment of physiological reactivity may impact both learning and decision-making on temporal stimulus attributes^20,29,119-123^; it is noteworthy that a high proportion (7/15, 47%) of bvFTD patients here failed the training phase of the temporal bisection task (see Table S4). It is further possible that pervasive shifts in hedonic valuation^31,124^ influenced sensitivity to these emotionally valenced sounds, particularly in the bvFTD and svPPA groups (compare group profiles in Fig. S2).

The neuroanatomical associations of altered perceived sound duration in the combined patient cohort (versus cognitively well controls) are in line with previous neuroimaging studies of supra-second temporal perception in the healthy brain^61,84,108^ and disturbed time perception following stroke.^125^ These associations lie within core neural networks previously implicated in the pathogenesis of AD and FTD^15,72,73,126^ and suggest candidate mechanisms for the modulation of subjective time in AD and FTD syndromes, separable by sound category and, within the category of human sounds, by emotional valence. For pleasant environmental sounds, overestimation of duration was associated with grey matter atrophy in the precuneus. This region has been identified in previous fMRI and electrophysiological studies as having a role in subjective time distortion^108^ and estimation of spatio-temporal magnitude^127,128^ and temporal intervals,^85,88,129^ particularly when engaging memory for previously learned temporal intervals and calibrated against internal bodily states. This may form part of a more general role in memory processes,^129,130^ in line with the status of the precuneus as a core component of the ‘default mode’ network.^131^ Arousal induced by environmental noises and other stimuli would modulate the self-referential calibration of stimulus duration in this neural circuitry.^132^

For human sounds, we found a more extensive and distinct set of neuroanatomical associations. It is plausible that the timing of human vocalizations should consume more neural resources than the processing of other sounds, on account of their greater behavioural salience: the associations here comprised lateral inferior parietal and insular cortices. These areas are closely related to the networks engaged in working memory and attention, which are intimately related to subjective time perception particularly where there is an output task,^84,89,128,133-135^ though these processes are dissociable.^59^ The anatomical differentiation by valence identified here is in line with previous evidence in older individuals,^136^ however it is likely that this additionally reflects stimulus-specific factors beyond valence. Supramarginal gyrus [an association here of underestimating pleasant human sound (laughter) duration] is integral to time perception^84,87^ but also involved in preparation to laugh and attribution of positive intent to heard laughter.^80,137,138^ Insula [an association here of underestimating unpleasant human sound (crying) duration] is key to marking out salient sensory events in time,^139^ by integrating incoming emotional sensory data with interoceptive signals.^4,89,108^ However, this region is also engaged in processing crying as an emotional signal, orienting responses to highly behaviourally relevant sounds and diverse other functions.^138^

From a clinical perspective, reduced sensitivity to temporal changes in AD and bvFTD and aberrant subjective time in nfvPPA and lvPPA may contribute to a diverse spectrum of neuropsychiatric symptoms, ranging from anxiety and depression to impulsivity, psychosis, disinhibition and social cognitive impairment.^140^ Such symptoms present significant challenges for diagnosis and management in FTD and AD syndromes.^15,141^ Potentially, disordered duration perception might impact any cognitive process reliant on accurate temporal coding: examples might include speech and prosody comprehension in nfvPPA and lvPPA,^100,109,142^ decision-making in bvFTD^143^ or auditory scene analysis in AD.^144^ Retained sensitivity to sound duration changes in svPPA (here, higher than in any other syndromic group) is likely to contribute to the temporal obsessionality with rigid punctuality and clockwatching that is a hallmark of this syndrome.^9,10,19,145,146^ In addition to novel diagnostic tests, time perception could present a novel target for management strategies based on behavioural interventions^140,147^ or pharmacotherapies exploiting retained dynamic neural plasticity.^148^

This study has several limitations that should inform further work. The interacting effects of sound semantic category with diagnosis and valence were observed in the absence of a significant, overarching interaction among all three factors: this likely reflects insufficient power, and indeed there is a need in future to engage larger cohorts. These should include a broader range of neurodegenerative syndromes, studied longitudinally. This is a particular issue given the wide individual variability within syndromic groups^8^ and will likely entail multi-centre collaboration. It will be important to include genetic cases: these have specified molecular pathology and can, in addition, allow psychophysical and physiological markers to be studied presymptomatically and prodromally,^149^ to establish how early temporal processing changes develop and how these evolve over the course of the illness.^9,14^ We also need to establish how alterations in time perception relate to other cognitive and behavioural symptoms in different neurodegenerative diseases. From a neuroscientific standpoint, the numerous factors that can influence subjective time perception should be studied systematically, using other paradigms besides temporal bisection^37^ with objective physiological measures of arousal and attention and a wider range of ecologically relevant sounds and multimodal stimuli.^150,151^ Elucidating the underlying neural mechanisms of subjective time perception will require functional neuroimaging studies, ideally employing techniques with high temporal resolution such as magnetoencephalography and examining network functional connectivity changes beyond the detection of grey matter atrophy (a substantial limitation of voxel-based morphometry). Given the interplay of dopaminergic and cholinergic pathways in interval timing,^152^ it would be of interest to combine such techniques with pharmacological manipulation, in neurodegenerative disorders with core neurotransmitter deficiencies such as AD and Lewy body disease. Moreover, future work should address aspects of temporal processing such as sub-second interval coding, motor reproduction and associated neural substrates^61,62,65^ beyond the focus of the present study.

## Conclusion

Subjective time is fundamental to how we experience the world but little explored in dementia. Our findings in AD and FTD syndromes suggest that time perception may be a novel paradigm for the neurobiological and clinical characterization of dementia syndromes: altered interval duration perception may be integrally linked to sensory signal and homeostatic processing in these diseases, in line with emerging physiological models of time perception that emphasize its embedding in sensory object perception.^153^ These findings should inform further research using naturalistic stimuli and study designs to define the clinical utility of temporal perception in dementia diagnosis and the development of behavioural and pharmacological interventions.

## Supplementary Material

fcaf496_Supplementary_Data

## Data Availability

The data that support the findings of this study are available on request from the corresponding author. The data are not publicly available because they contain information that could compromise the privacy of research participants. Related code is available on OSF in the project specific repository: https://doi.org/10.17605/OSF.IO/W2JRS.
