# Supplementary material for: Subjective time perception in dementia: a behavioural and neuroanatomical analysis
Source: Brain Commun. 2025 Dec 30;8(1):fcaf496. doi: 10.1093/braincomms/fcaf496 (PMC12782024; doi:10.1093/braincomms/fcaf496)
Supplement: fcaf496_Supplementary_Data [file fcaf496_supplementary_data.zip › Supplementary_material.docx]

**Supplementary Material.**

**Subjective time perception in dementia: a behavioural and neuroanatomical analysis, by M-C Requena-Komuro et al.**

# Auditory duration and recognition control tasks

The tone duration categorisation task followed a similar procedure to the main temporal bisection task. Participants were instructed to categorise a sound (here a pure tone) into a “short” or “long” interval. Pure tones were created using the Generate module in Audacity, by specifying the following characteristics: sine waveform, frequency (in Hz), amplitude of 0.8, and duration of 2 or 5 seconds. All sound files were then rms-fixed at the same intensity level as the sound files for the temporal bisection task and exported as wav files. Participants were first asked to complete eight practice trials (four different frequencies – 200, 300, 1000, 1100 Hz – lasting either 2 or 5 seconds) presented in randomised order. A minimum score of 75% (6 correct responses) was required to complete the experimental phase. The experimental phase consisted of twelve or eight trials (tones chosen among the following frequencies - 400, 500, 600, 700, 800, 900 Hz – lasting either 2 or 5 seconds) where participants categorised the sounds as “short” or “long” without any feedback. Participants provided their answers in the same way as for the temporal bisection task.

To account for differences in auditory semantic knowledge as a potentially confounding factor, patients further completed a sound recognition task for the categories of auditory stimuli used in the main experimental task (see **Figure S4**). In this task, patients were asked to choose the picture that matched the sound among a choice of four different pictures (the target and three foils), each representing one of the four sound categories. 16 trials (two examples for each of the eight sound types) were administered in randomised order and all sound files were taken from the main timing experiment.

# Assessment of peripheral hearing function

Participants who were assessed in person underwent pure tone audiometry using a dual-channel GSI Audiostar Pro audiometer and following protocol outlined by the British Society of Audiology (<https://www.thebsa.org.uk/wp-content/uploads/2018/11/Recommended-Procedure-Pure-Tone-Audiometry-August-2018-FINAL.pdf>). Participants sat in a quiet room and were tested on each ear separately, starting with their better ear. To determine their hearing thresholds, they listened to pure tones of varying frequencies (starting at 1000Hz, then 2000Hz, 4000Hz, 8000Hz, then 500Hz, 250Hz, and finally 1000Hz) over descending intensity levels. Specifically, at each frequency, they were asked to indicate (verbally or using a gesture) if they could hear the sound, starting at a clearly audible level (usually 50 dB HL (decibel hearing level)). If they could, the level was decreased in steps of 10 dB until they could not, at which point the level was increased by steps of 5 dB, until they could hear it again. This procedure was repeated three times to establish the mean threshold at that frequency. For each participant, a composite hearing threshold was then created by calculating the mean threshold across all frequencies in the better ear.

# Musical experience questionnaire

Extensive musical experience may significantly improve timing performance (Ehrle & Samson, 2005; Rammsayer & Altenmüller, 2006). Accordingly, participants were asked to complete a music questionnaire that covered previous and current musical training and which we recently validated (van’t Hooft et al., 2024). The following scoring procedure was applied:

Score 0: never played an instrument or engaged in singing, either formally or informally

Score 1: played an instrument or engaged in singing < 1 year, either formally or informally

Score 2: > 2 years’ instrument/singing experience but no grade

Score 3: reached grade 3 or 4 on an instrument/singing, or at least 10 years’ experience without a grade

Score 4: reached grade 7 or 8 on an instrument/singing

# Mood questionnaire

To ensure participants’ performance was not affected by poor mental health (especially due to the COVID-19 pandemic), all participants tested during that period (from February 2021 to December 2021) were asked to fill in a mood questionnaire prior to completing any experimental session. The questionnaire was adapted from the Immediate Mood Scaler (Nahum et al., 2017) and included four questions for which responses were scored on a 5-point Likert scale covering the following dimensions: Happy-Sad, Energetic-Tired, Interested-Bored, and Peaceful-Anxious. A composite score (sum of ratings over all four dimensions, scored out of 20) was calculated for further analysis.

# Secondary analysis of temporal bisection task data: valence reassignment

Some patients produced valence ratings that did not fit pre-defined sound categories. We addressed this in a secondary analysis, in which the corresponding sounds were reassigned to the ‘correct’ category for each individual (for example, if a patient found angle grinder sounds pleasant, for that person those sounds were assigned to the ‘environmental pleasant’ rather than the ‘environmental unpleasant’ category). The reassignments made across the patient cohort are summarised in **Table S2**. As a result of these changes, one svPPA patient was excluded from the secondary analysis sample because they rated all sounds as pleasant; eight patients had one ‘missing’ sound category (two environmental pleasant, two environmental unpleasant, three human pleasant, one human unpleasant) but were maintained in the secondary analysis sample.

Psychometric curves were estimated, corresponding bisection point and Weber’s ratio vales were calculated and a statistical analysis was performed, all following the same procedures as for the primary analysis described in main text Methods. Results are summarised in **Table S3**.

# Training phase of temporal bisection experiment

Details of patients failing the training phase of the temporal bisection task are presented in Table S4. The bvFTD group had the highest number (seven); three also failed the pure tone duration task, suggesting that they were particularly impaired on temporal judgments. The remaining four had an average tone duration score lower than those who completed the full temporal bisection task (83.3% vs 96.9%; see Table 2). Performance on the sound recognition task was similar (95.5% vs 97.8%; see Table 2). Only one of the four AD patients who failed the training phase of the temporal bisection task also failed the pure tone duration task; average tone duration scores of the remaining three were similar to those who completed the full temporal bisection task (93.3% vs 94.1%; see Table 2), while average sound recognition score was lower (81.3% vs 98.9%; see Table 2).

# Brain image acquisition and pre-processing

Volumetric T1 MR brain images were acquired on a Siemens Prisma 3T MRI scanner using a 32-channel phased array head-coil and following a sagittal 3D magnetization-prepared rapid-gradient echo T1-weighted volumetric brain MR sequence (echo time/repetition time/inversion time respectively 2.9/2200/900ms, dimensions 256 × 256 × 208, voxel volume of 1.1 × 1.1 × 1.1mm). Prior to pre-processing, each scan was examined for quality control. Data from 27 patients (two AD, seven bvFTD, nine svPPA, four nfvPPA, five lvPPA) were entered into the VBM analysis (scans were not available for the other patients due to remote testing).

Pre-processing of brain images was performed using the New Segment and DARTEL toolboxes in SPM12 (www.fil.ion.ucl.ac.uk/spm/software/spm12/), following an optimized protocol (Ridgway et al, 2008). Normalization, segmentation, and modulation of grey and white matter images were carried out using default parameter settings. Grey matter images were subsequently smoothed using a 6mm full width-at-half-maximum Gaussian kernel. For each patient, total intracranial volume was calculated by summing grey matter, white matter, cerebrospinal fluid volumes after segmentation of these tissue types. A study-specific template brain image was created by warping all bias-corrected native space brain images to the final DARTEL template and calculating the average of the warped brain images.

# Supplementary references

Ehrle, N., & Samson, S. (2005). Auditory discrimination of anisochrony: influence of the tempo and musical backgrounds of listeners. *Brain Cogn, 58*(1), 133-147. doi:10.1016/j.bandc.2004.09.014

Nahum, M., Van Vleet, T. M., Sohal, V. S., Mirzabekov, J., Rao, V. R., Wallace, D. L., . . . Change, E. F. (2017). Immediate Mood Scaler: Tracking Symptoms of Depression and Anxiety Using a Novel Mobile Mood Scale. *JMIR Mhealth Uhealth, 5*(4), e44. doi:10.2196/mhealth.6544

Rammsayer, T. H., & Altenmüller, E. (2006). Temporal Information Processing in Musicians and Nonmusicians. *Music Percept, 24*(1), 37-48. doi:10.1525/mp.2006.24.1.37

van’t Hooft, J. J., Benhamou, E., Albero Herreros, C., Jiang, J., Levett, B., Core, L. B., . . . Warren, J. D. (2024). Musical experience influences socio-emotional functioning in behavioural variant frontotemporal dementia. *Frontiers in Neurology, 15*. doi:10.3389/fneur.2024.1341661

Table S1. Example sounds for the temporal bisection task

| Sound file number | Sound condition | Sound example |
| --- | --- | --- |
| 1 | Environmental unpleasant | Angle grinder |
| 2 |  | Car horn |
| 3 | Environmental pleasant | Brook |
| 4 |  | River |
| 5 | Human unpleasant | Male crying |
| 6 |  | Female crying |
| 7 | Human pleasant | Male laughing |
| 8 |  | Female laughing |

All selected sound files are 3.5 seconds long and have been created following the procedures described in Methods. All sound files are distributed under the CC BY license (except for sound file #6, which is available under the CC BY-NC license). Credit goes to (listed in the order of sound file number; all correspond to usernames from freesound.org): Benboncan, ikbenraar, tim.kahn, CLaforet, qubodup, Idalize, sheblum. No attribution is required for the male laughing sound file obtained from soundsnap.com.

**Table S2. Summary of valence reassignments based on individual participant valence ratings**

| **Sound** | **Angle grinder** | **Car horn** | **River** | **Brook** | **Male crying** | **Female crying** | **Male laughter** | **Female laughter** |
| --- | --- | --- | --- | --- | --- | --- | --- | --- |
| **AD** | 1 | 0 | 0 | 0 | 0 | 1 | 4 | 2 |
| **lvPPA** | 0 | 0 | 0 | 0 | 0 | 0 | 0 | 0 |
| **nfvPPA** | 2 | 0 | 1 | 0 | 0 | 0 | 0 | 1 |
| **svPPA** | 4 | 2 | 2 | 3 | 1 | 1 | 0 | 0 |
| **bvFTD** | 2 | 3 | 1 | 0 | 1 | 0 | 2 | 1 |

Total numbers of reassignments made are indicated for each diagnostic group (five AD, four nfvPPA, eight svPPA, five bvFTD) and sound category (note that sometimes more than one change was made for an individual participant). AD, patient group with typical Alzheimer’s disease; bvFTD, patient group with behavioural variant frontotemporal dementia; Controls, healthy control group; lvPPA, patient group with logopenic variant primary progressive aphasia; nfvPPA, patient group with nonfluent/agrammatic primary progressive aphasia; svPPA, patient group with semantic variant primary progressive aphasia.

**Table S3. Results of secondary analysis of temporal bisection task data using adjusted sound valence ratings**

| **Diagnosis** | **Healthy controls** | | **AD** | | **lvPPA** | | **nfvPPA** | | **svPPA** | | **bvFTD** | |
| --- | --- | --- | --- | --- | --- | --- | --- | --- | --- | --- | --- | --- |
| **Sound condition** | Env | Hum | Env | Hum | Env | Hum | Env | Hum | Env | Hum | Env | Hum |
| Bisection point | | | | | | | | | | | | |
| Unpleasant | 3.3 (0.4) | 4.3 (0.5) | 3.1 (0.6) | 4.5 (0.5) | 3.3 (0.5) | 4.7 (0.4) | 3.0 (0.6) | 4.7 (0.6) | 3.4 (0.5)  ^n-2^ | 4.3 (0.5)  ^n-2^ | 3.4 (0.5)  ^n-1^ | 4.5 (0.5) |
| Pleasant | 3.6 (0.4) | 4.1 (0.4)  ^n-3^ | 3.3 (0.5) | 4.2 (0.4)  ^n-2^ | 3.3 (0.5) | 4.4 (0.5) | 3.3 (0.8) | 4.3 (0.7) | 3.4 (0.3)  ^n-3^ | 4.0 (0.3)  ^n-1^ | 3.6 (0.5) | 4.4 (0.7)  ^n-1^ |
| **Weber’s ratio** | | | | | | | | | | | | |
| Unpleasant | 0.20 (0.07) | 0.18 (0.06) | 0.28 (0.08) | 0.22 (0.11) | 0.25 (0.08) | 0.24 (0.08) | 0.22 (0.09) | 0.22 (0.08) | 0.19 (0.05)  ^n-2^ | 0.21 (0.05)  ^n-2^ | 0.22 (0.09)  ^n-1^ | 0.20 (0.06) |
| Pleasant | 0.23 (0.08) | 0.22 (0.07)  ^n-3^ | 0.28 (0.11) | 0.28 (0.13)  ^n-2^ | 0.25 (0.07) | 0.24 (0.10) | 0.27 (0.11) | 0.26 (0.10) | 0.17 (0.08)  ^n-3^ | 0.22 (0.08)  ^n-1^ | 0.30 (0.19) | 0.28 (0.10)  ^n-1^ |

The table presents the results of the secondary analysis after re-categorising sounds as ‘pleasant’ or ‘unpleasant’ based on individual participant sound valence ratings (see also **Table 3**). Differences from the primary analysis are underlined and the number of missing participants as a result of sound reassignment coded as n-x, where x is the number of ‘missing’ participants. **For bisection point**, as for the primary analysis, the linear mixed model revealed a main effect of sound semantic category and significant two-way interactions between semantic category and diagnosis, and semantic category and valence (all p < 0.001); post-hoc comparisons were also similar, except the svPPA group no longer overestimated the duration of human sounds compared to bvFTD patients (p > 0.05). **For Weber’s ratio,** as for the primary analysis, the linear mixed model revealed significant main effects of diagnosis (p = 0.0178) and valence (p = 0.0092); however, in contrast to the primary analysis, there was no significant interaction between sound semantic category and diagnosis (p > 0.05); post-hoc comparisons were also similar, except the bvFTD group no longer had a lower discrimination sensitivity compared to healthy controls. AD, patient group with typical Alzheimer’s disease; bvFTD, patient group with behavioural variant frontotemporal dementia; Env, environmental sound category; Hum, human sound category; lvPPA, patient group with logopenic variant primary progressive aphasia; nfvPPA, patient group with nonfluent/agrammatic primary progressive aphasia; svPPA, patient group with semantic variant primary progressive aphasia.

Table S4. Characteristics of patients failing the training phase of the temporal bisection task

| **Characteristic** | **AD** | **lvPPA** | **nfvPPA** | **bvFTD** |
| --- | --- | --- | --- | --- |
| **Demographic** | | | | |
| N (male/female) | 4 (3/1) | 1 (1/0) | 2 (2/0) | 7 (7/0) |
| Age (years) | 66.3 (7.8) | 74.0 (N/A) | 76.0 (4.2) | 66.7 (6.0) |
| Handedness (right/left) | 4/0 | 1/0 | 2/0 | 6/1 |
| Education (years) | 17.0 (2.2) | 12.0 (N/A) | 14.5 (3.5) | 15.1 (3.0) |
| Symptom duration (years) | 5.5 (2.1) | 3.0 (N/A) | 3.5 (0.7) | 5.0 (2.7) |
| T-MMSE (/27) | 11.3 (5.0) | 22.0 (N/A) | 15.5 (13.4) | 17.8 (3.9) ^n-1^ |
| **Performance on control tasks** | | | | |
| Pure tone duration score (%) | 93.3 (6.1)  ^n-1^ | 100 (N/A) | N/A | 83.3 (18.2)  ^n-3^ |
| Sound recognition score (%) | 81.3 (22.5)  ^n-1^ | 100 (N/A) | 31.3 (N/A)  ^n-1^ | 95.5 (6.9) |

Mean (standard deviation) values are shown unless otherwise specified. Standard deviations could not be calculated for the single participant with lvPPA. Missing data (either because partipants failed the training phase of the control task, or because participants did not understand the task instructions) are coded n-x, where x is the number of ‘missing’ participants. There are no data available on the pure tone duration categorisation task for nfvPPA patients because both discontinued the testing session. AD, patient group with typical Alzheimer’s disease; bvFTD, patients with behavioural variant frontotemporal dementia; lvPPA, patient with logopenic variant primary progressive aphasia; N/A, not available; nfvPPA, patients with nonfluent/agrammatic primary progressive aphasia; T-MMSE, telephone-Mini-Mental State Examination score.

Figure S1. Group mean valence ratings for all sound stimuli


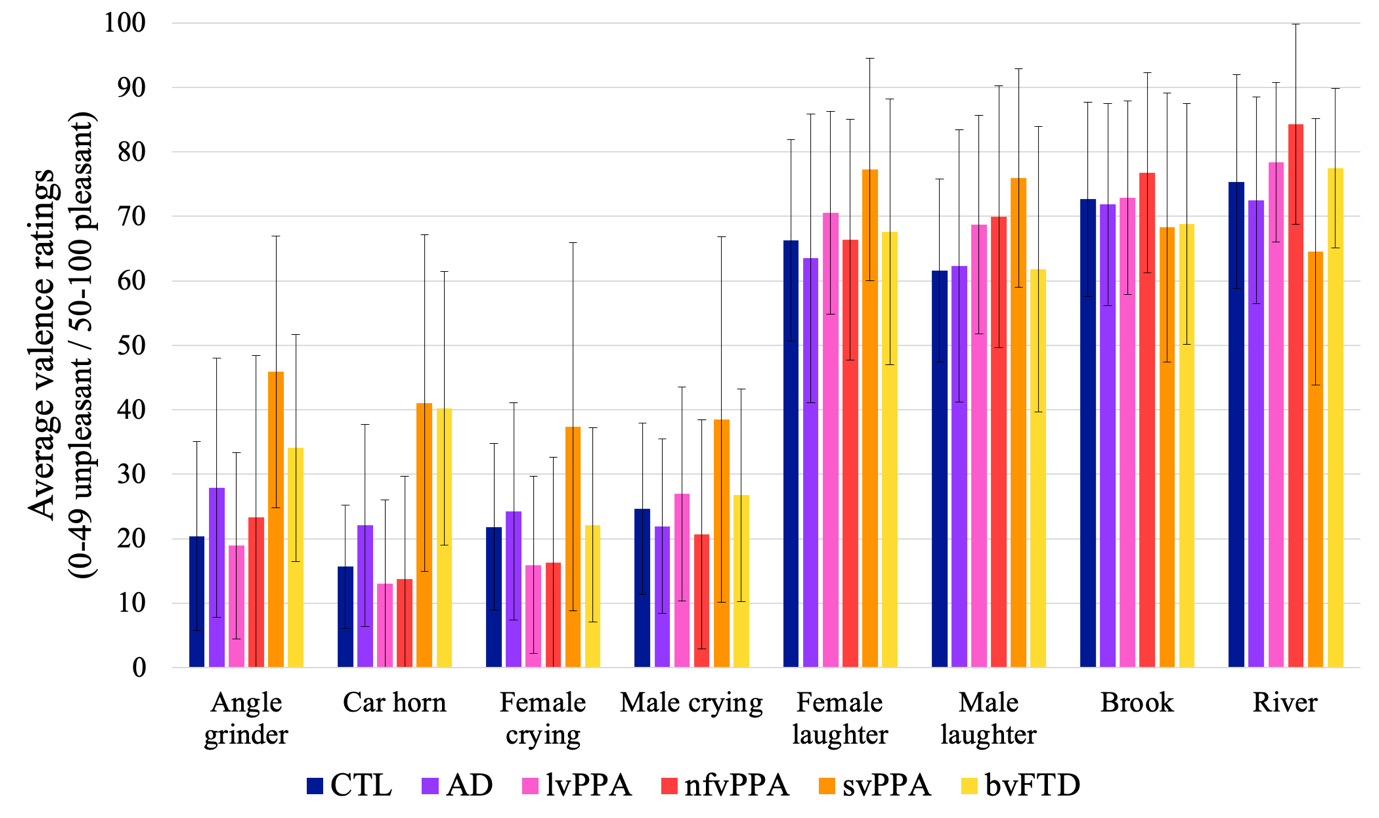


Sound valence ratings were obtained by asking participants (n = 70) to use the mouse to match their subjective rating to a position on a sliding scale from “extremely unpleasant” to “extremely pleasant”. These positions were then converted to scores (0-49 corresponding to unpleasant, 50-100 corresponding to pleasant). Mean values across participants from each group have been calculated for each sound, and error bars indicate standard deviations. In general, the sound stimuli were found to fit their pre-defined valence categories: sounds that were pre-defined as unpleasant (female and male crying, angle grinder, car horn) were on average rated as unpleasant by each participant group, while sounds pre-defined as pleasant (female and male laughing, brook, river) were on average rated as pleasant by each participant group. For seven healthy control participants, the mean valence assigned to at least one sound did not fit the pre-defined valence category (five for laughter, one for river, and one for angle grinder); these sounds were reassigned to the appropriate individual valence category prior to analysis. To evaluate any potential between-group differences in valence ratings, a linear regression model was implemented, with rating as the dependent variable and both sound identity (one of the eight sounds) and group as the independent variables. Residuals were not normal even after removing one outlier participant and attempting transformation (logarithmic, square root, and inverse). Bootstrapping was therefore carried out with 1000 replications and sampling with replacement. The linear regression yielded a significant main effect of sound identity (X2 = 856.76, df = 7, p<0.0001) and a significant main effect of group (X2 = 12.17, df = 5, p=0.0325). Exploratory post-hoc comparisons revealed that svPPA patients produced higher valence ratings compared to healthy control participants across all sounds (z=3.01, p = 0.039). A significant interaction between sound identity and group was also found (X2 = 54.63, df = 35, p=0.0184). Corresponding post-hoc comparisons revealed that svPPA patients found angle grinder and car horn sounds more pleasant than did healthy controls (z=4.29, p<0.001 and 3.27, p=0.016 respectively) and nfvPPA patients (z=-3.34, p=0.012 and -2.96, p=0.046 respectively). bvFTD patients also found car horn sounds more pleasant than did healthy controls (z=3.04, p=.035) and nfvPPA patients (z=-2.94, p=0.049). AD, patient group with typical Alzheimer’s disease; bvFTD, patient group with behavioural variant frontotemporal dementia; CTL, healthy control group; lvPPA, patient group with logopenic variant primary progressive aphasia; nfvPPA, patient group with nonfluent/agrammatic primary progressive aphasia; svPPA, patient group with semantic variant primary progressive aphasia.

**Figure S2.** K-density plots of valence ratings for all participant groups


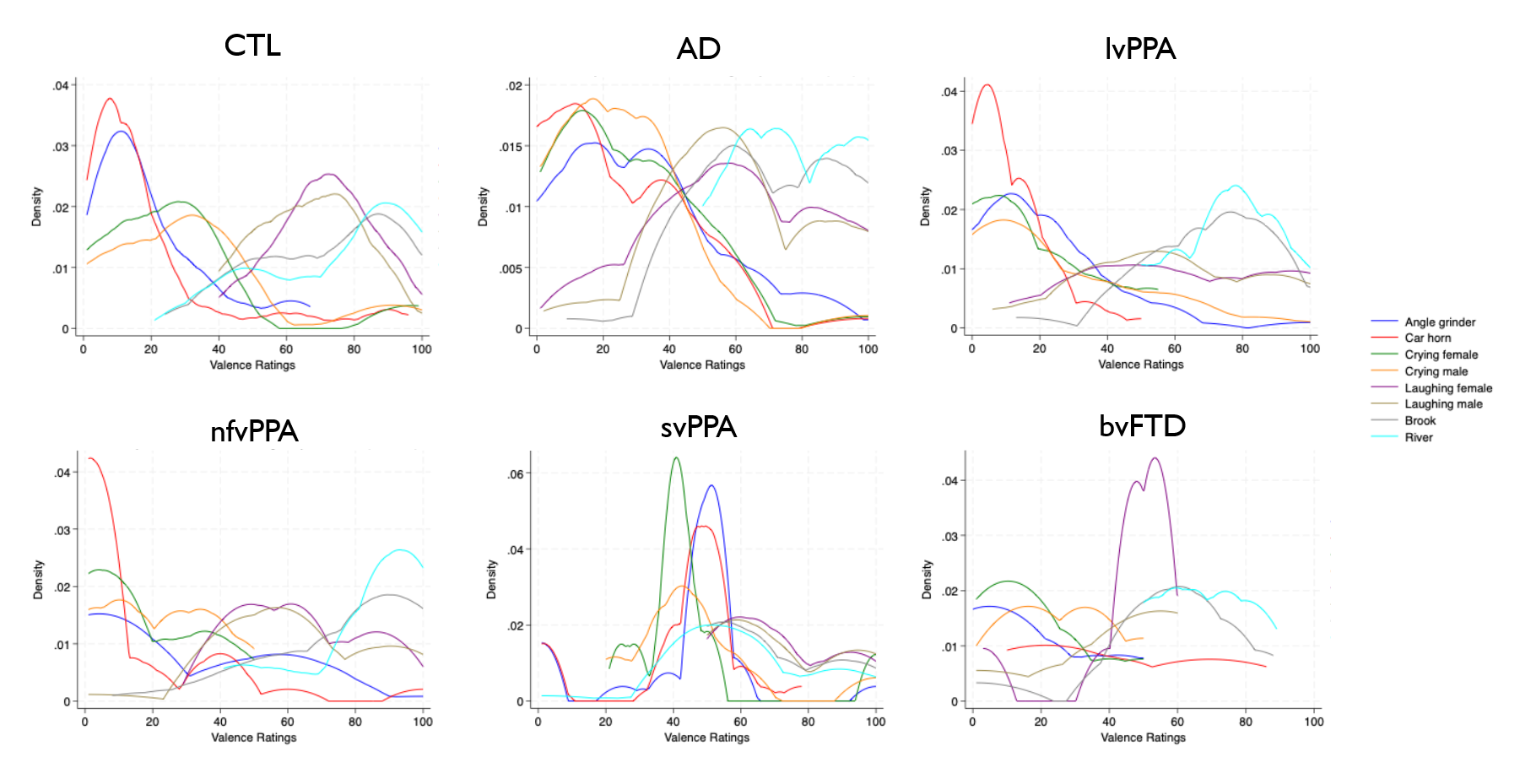


k-density plots of valence ratings for participant group and each sound, color-coded as indicated in the legend. AD, patient group with typical Alzheimer’s disease; bvFTD, patient group with behavioural variant frontotemporal dementia; CTL, healthy controls; lvPPA, patient group with logopenic variant primary progressive aphasia; nfvPPA, patient group with nonfluent/agrammatic primary progressive aphasia; svPPA, patient group with semantic variant primary progressive aphasia.

**Figure S3**. Flowchart of patient sample at each stage of this study


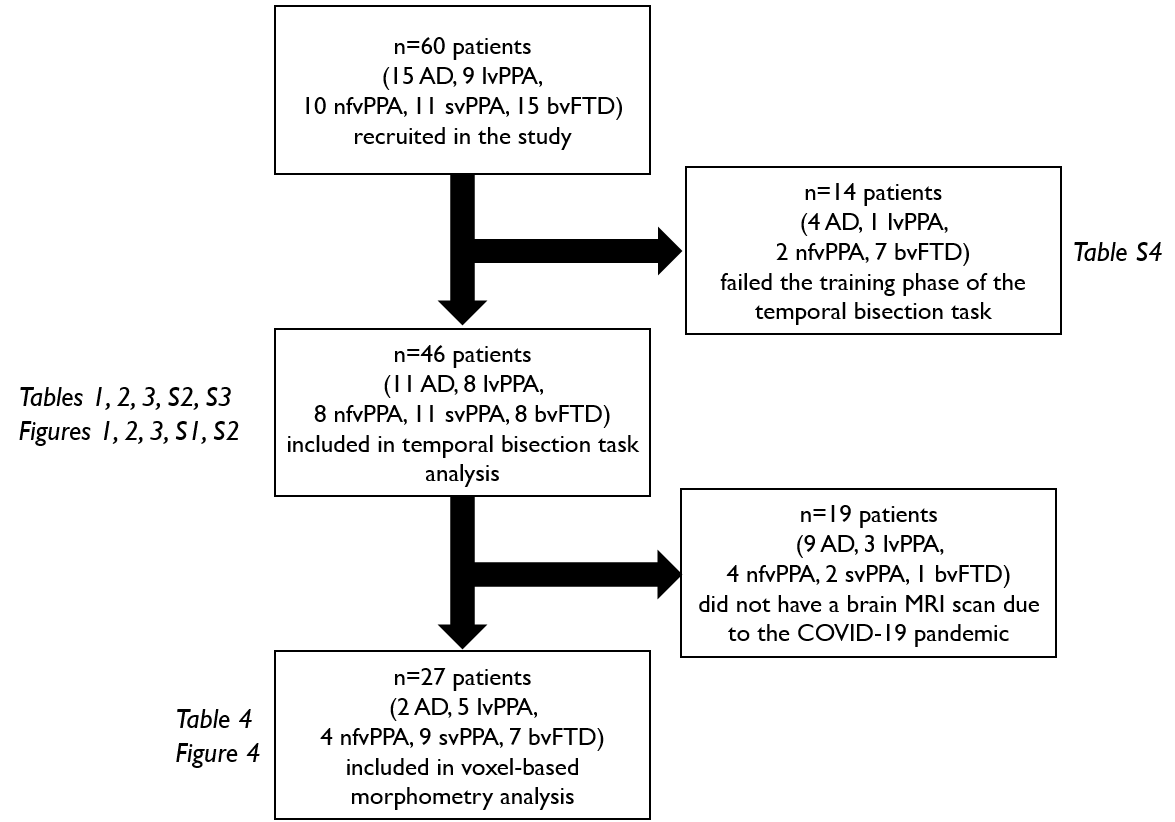


Flowchart indicating the patient sample recruited and analysed at each stage of this study, split by diagnosis. AD, patient group with typical Alzheimer’s disease; bvFTD, patient group with behavioural variant frontotemporal dementia; lvPPA, patient group with logopenic variant primary progressive aphasia; MRI, magnetic resonance imaging; nfvPPA, patient group with nonfluent/agrammatic primary progressive aphasia; svPPA, patient group with semantic variant primary progressive aphasia.

**Figure S4**. Sound recognition control task: trial example


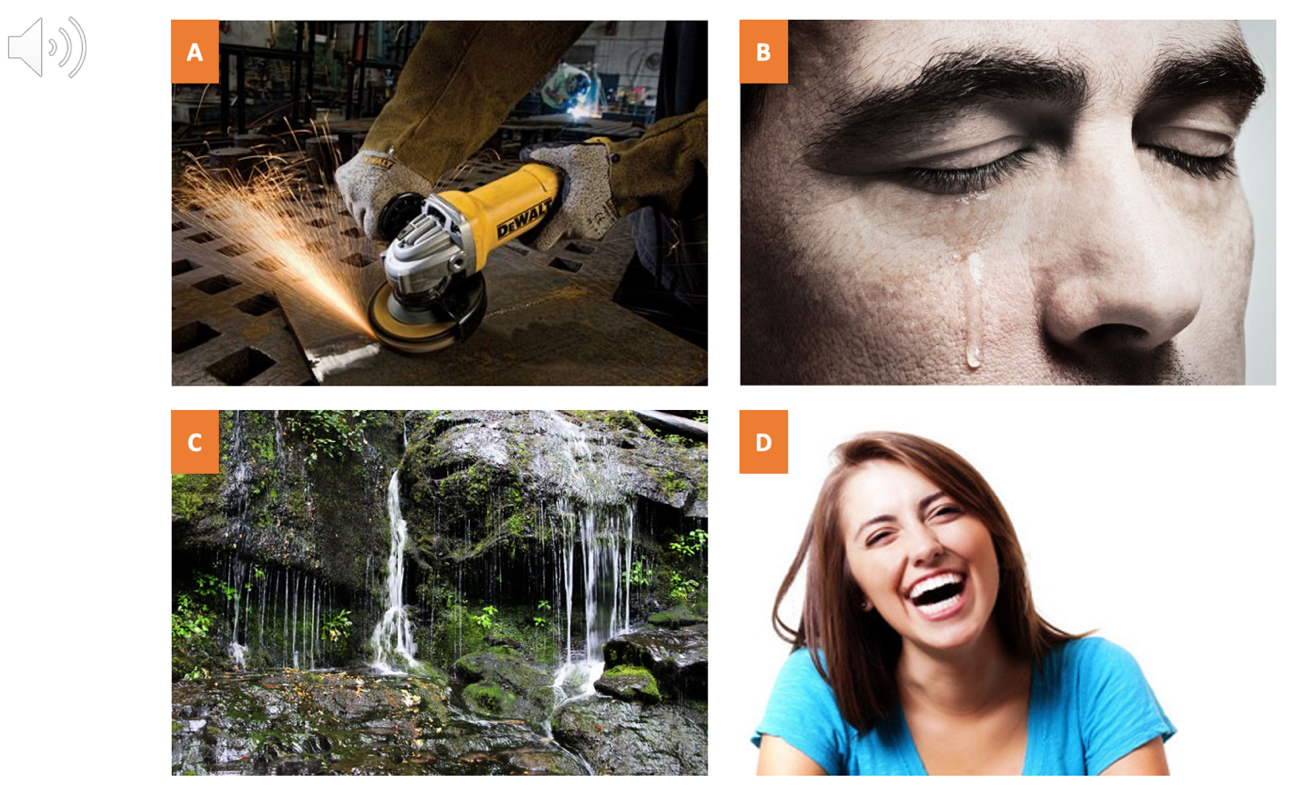


On each trial, participants listened to a sound and were asked to choose the picture that matched the sound among a choice of four, each picture representing one experimental auditory condition (for example here: **A**, environmental unpleasant; **B**, human unpleasant; **C**, environmental pleasant; **D**, human pleasant). The screen positions of target and foil pictures were counterbalanced from trial to trial, such that they were equally displayed among the four locations across trials. The order of presentation of the sounds was also pseudo-randomised, to avoid two examples of the same sound category being played on consecutive trials.

Figure S5. Pre-specified neuroanatomical regions of interest for the VBM analysis


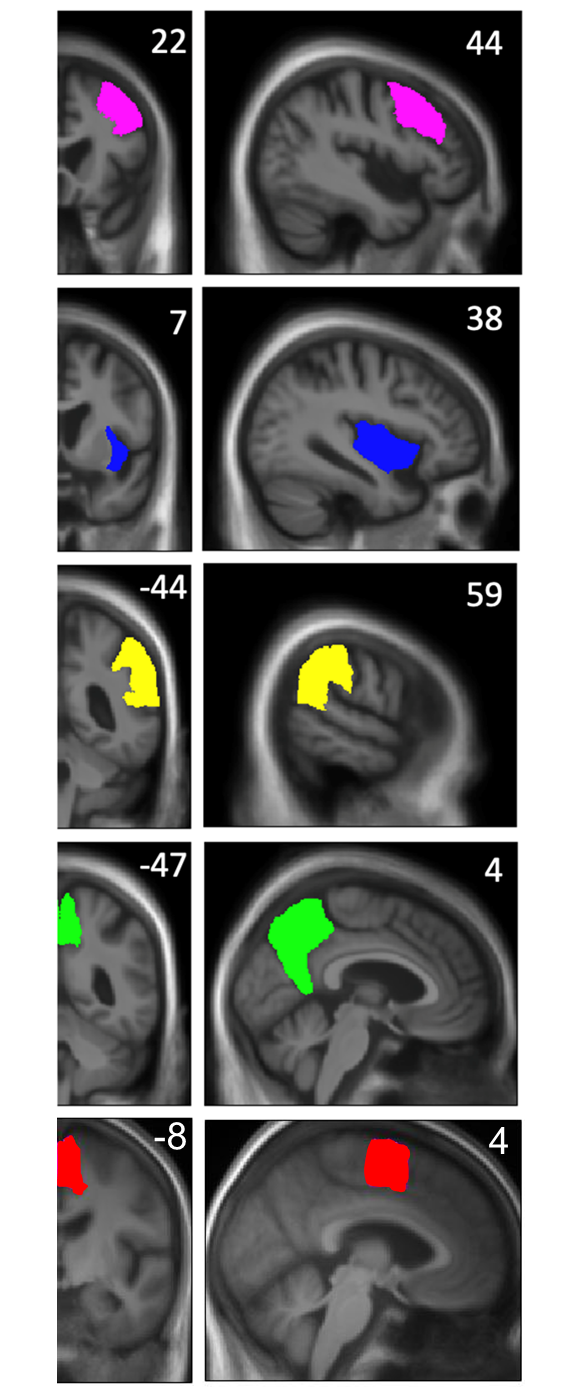


Coronal (left) and sagittal (right) views of the regions of interest used in the voxel-based morphometry analysis during small volume correction for multiple voxel-wise comparisons (see text for details). Regions have been overlaid on the group mean structural brain magnetic resonance image in MNI (Montreal Neurological Institute) space, coordinates (mm) of the plane of each section are indicated. Magenta: middle frontal gyrus; blue: insular cortex; yellow: inferior and superior parietal lobule; green: precuneus; red: supplementary motor area.
